# Targeting neutrophil‐driven immunosuppression: A strategy to overcome immune checkpoint inhibitor resistance

**DOI:** 10.1002/ctm2.70582

**Published:** 2026-01-05

**Authors:** Ying Ning, Ke Lei, Xinyan Gao, Yan Kong, Yuping Shan, Tian Tian, Zhumei Cui, He Ren

**Affiliations:** ^1^ Department of Obstetrics and Gynecology The Affiliated Hospital of Qingdao University Qingdao China; ^2^ Center of Tumor Immunology and Cytotherapy Medical Research Center The Affiliated Hospital of Qingdao University Qingdao China

**Keywords:** immune checkpoint inhibitors, neutrophils, resistance, mechanism, combination therapy

## Abstract

Immune checkpoint blockade (ICB) has revolutionized tumour therapy by relieving immunosuppression and restoring effector T cell cytotoxicity. However, its clinical utility is constrained by low response rates and acquired resistance. Tumour‐associated neutrophils (TANs), key players in tumour immunoregulation, have emerged as critical mediators of ICB responsiveness and resistance, highlighting the therapeutic potential of combining TAN‐targeted strategies with immune checkpoint inhibitors (ICIs). This review systematically synthesizes current knowledge of neutrophils in ICB resistance from several dimensions: (1) clinical indicators of neutrophils, such as the neutrophil‐to‐lymphocyte ratio (NLR) and tissue TANs abundance, as predictors of ICI response and patients prognosis; (2) multifaceted TAN‐involved resistance mechanisms, including direct T cell inhibition, antigen presentation impairment, function modulation of other immune cells, promotion of tumour angiogenesis, and elevation of tumour mutation burden (TMB); (3) combination therapeutic strategies targeting TAN generation/ exhaustion, recruitment, phenotypic polarization, activation, proangiogenic functions, and neutrophil extracellular traps (NETs), along with progress in related clinical trials. Combinatorial approaches integrating TAN‐targeted therapies with ICIs hold substantial promise for overcoming resistance by reshaping the immune microenvironment. Elucidating neutrophil‐mediated resistance mechanisms and optimizing combination strategies will pave the way for precision tumour immunotherapy.

**Key points:**

- TANs drive ICI resistance via antitumour immune remodelling, angiogenesis promotion, and elevation of tumour mutation burden
- Neutrophil biomarkers (e.g., NLR, TAN abundance) show strong predictive value for ICI response and prognosis.
- Targeting TAN recruitment, polarization, function and NETosis represents a promising strategy to overcome ICI resistance.
- Numerous clinical trials are evaluating combination therapies targeting neutrophils to enhance immunotherapy efficacy.

## Graphical abstract

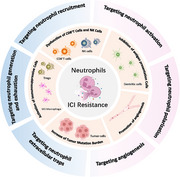

## 1 INTRODUCTION

Immune checkpoint inhibitors (ICIs), by targeting key immunosuppressive pathways, markedly improve survival outcomes across multiple malignancies and represent landmark advances in tumour immunotherapy. Currently, ICIs have been approved for the treatment of over a dozen solid tumours.^1^ However, their clinical application remains challenged by low response rates (primary resistance) and disease progression following initial response (acquired resistance). Clinical data indicate that only 20–30% of patients exhibit durable responses to ICI monotherapy,^2^ while over 50% of initial responders eventually experience disease progression due to acquired resistance.^2^ The complexity of resistance mechanisms stems from both tumour‐intrinsic heterogeneity and dynamic microenvironmental regulation, making the precise elucidation of these mechanisms and development of reversal strategies a focal point of current research.

The mechanisms underlying tumour resistance to ICIs are multifaceted, stemming from both tumour‐intrinsic heterogeneity and dynamic interactions with the tumour microenvironment (TME). Research in this field has traditionally focused on cytotoxic T cells as the primary effector cells of ICIs and on regulatory T cells as key mediators of immune tolerance.^3^ Among innate immune cells, natural killer (NK) cells, which support antitumour immunity, and tumour‐associated macrophages (TAMs), which assist in immunosuppression, have also garnered considerable attention for their impact on ICI efficacy.^4^ However, emerging evidence underscores a pivotal and distinct function for neutrophils, the most abundant effector leukocytes of the innate immune system, in modulating resistance to ICI therapy.^5^

Neutrophils constitute 50–70% of human peripheral blood leukocytes.^6^ Their antimicrobial mechanisms include degranulation, phagocytic activity, and the generation of neutrophil extracellular traps (NETs).^7^ Physiologically, neutrophils experience a short lifespan (circulating half‐life <24 h, with tissue residence spanning several days) and their activation and recruitment are tightly regulated to prevent potential damage from their cytotoxic activities to normal tissues.^8^ In pathological contexts, however, neutrophils respond to diverse stimuli, infiltrating sites of injury or disease. Sustained neutrophil infiltration can lead to chronic inflammation and tissue damage, often facilitating tumour initiation, progression and drug resistance.^9^ These neutrophils, upon pathological activation within the tumour microenvironment, are alternatively designated polymorphonuclear myeloid‐derived suppressor cells (PMN‐MDSCs).^10^ Several neutrophil‐derived biomarkers, notably the peripheral blood neutrophil‐to‐lymphocyte ratio (NLR) and tissue TAN abundance, have been identified as closely related to the efficacy of ICI therapy. These findings have shifted neutrophils from traditional ‘bystanders’ to central players in research on resistance mechanisms of tumour immunotherapy, particularly for ICIs.

**FIGURE 1:**
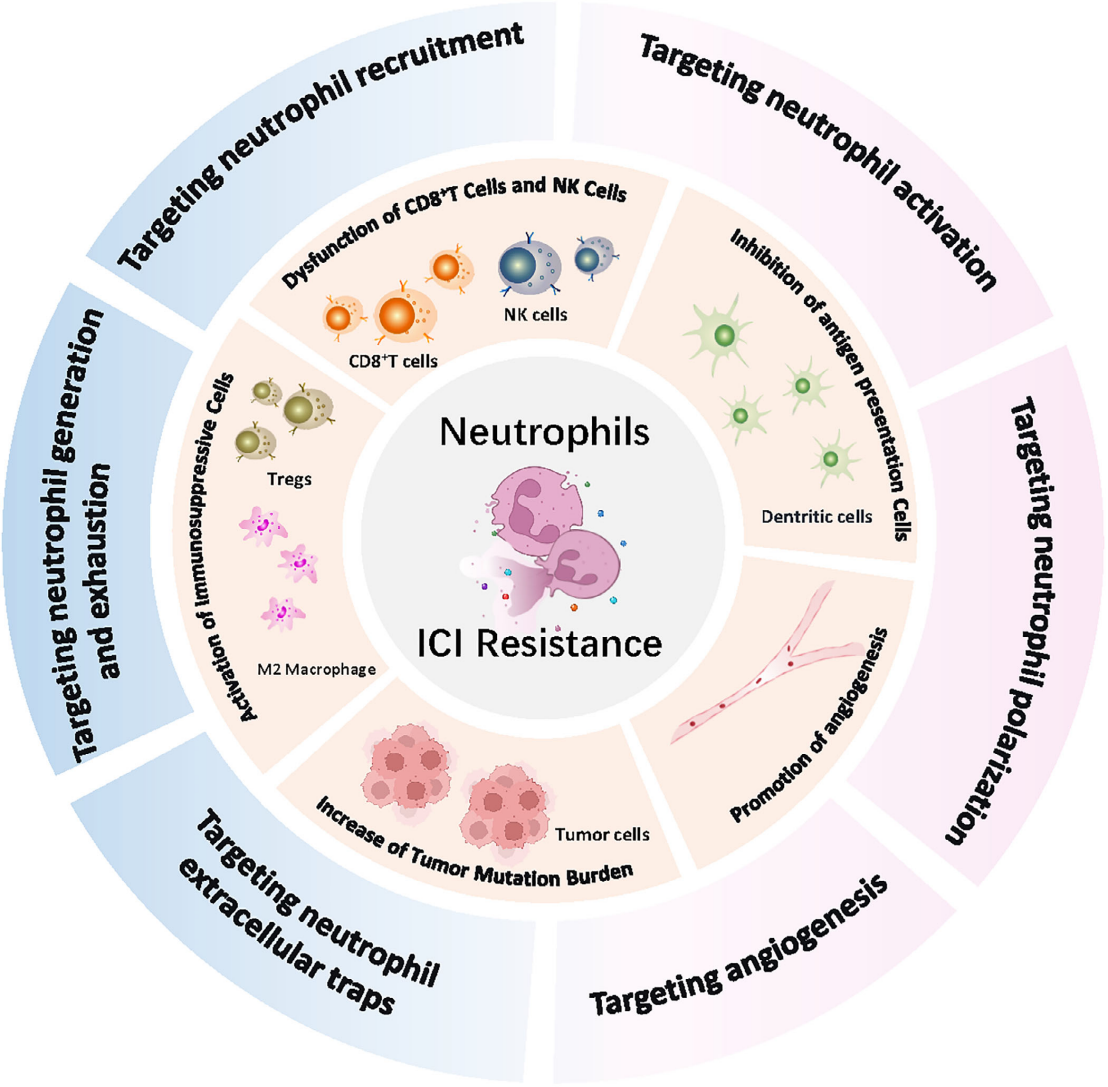
Tumour‐associated neutrophils (TANs) drive resistance to immune checkpoint inhibitors (ICBs) through multiple mechanisms. Targeting TANs reshapes the tumour immune microenvironment and enhances antitumour immunity, making this therapy a promising combination strategy to overcome ICI resistance.

This review summarizes the predictive value of neutrophil‐related clinical indicators for ICI resistance, systematically reviews the key mechanisms by which TANs mediate ICI resistance, and discusses the clinical trials of neutrophil‐targeting drugs combining with ICIs (Figure 1). It highlights the promising potential of such combination strategies in tumour immunotherapy.

### 1.1 Clinical indicators related to neutrophils and ICI therapy

Neutrophil‐related clinical indicators have emerged as critical predictors of patient response to ICIs and prognostic assessment. The NLR serves as the most readily accessible peripheral blood biomarker for unfavourable outcomes in numerous solid malignancies. (Table S1).^11^, ^12^ In patients receiving ICI therapy, a high baseline NLR correlates significantly with shortened survival period, and may partially reflect treatment response rates of ICIs.^13^, ^14^, ^15^ For example, in gastric cancer (GC) cohorts, pre‐treatment NLR levels effectively stratify patient subgroups more likely to benefit from PD‐1 inhibitors.^16^ Similarly, monitoring NLR during ICI treatment in metastatic or recurrent HNSCC facilitates early identification of patients at risk of treatment failure.^17^ These results highlight the detrimental role of neutrophils in modulating ICI efficacy.

Compared with peripheral blood markers, TANs, by directly participating in TME regulation, exhibit more intimate and complex associations between their phenotypic heterogeneity, infiltration abundance and clinical outcomes.^18^ Early preclinical studies in mouse models proposed a functional dichotomy of TANs into pro‐tumour N2‐type and anti‐tumour N1‐type.^19^ In gastric cancer, N2‐TANs enhance tumour invasive capacity by releasing pro‐tumour exosomes.^20^ Conversely, neutrophils can also exert anti‐tumour activity and prevent early lung metastasis in mouse models of lung cancer.^21^ However, the simplistic N1/N2 classification may insufficiently capture the functional plasticity of TANs, given their phenotypic diversity in TME. Most current clinical studies analyze the impact of overall TAN abundance on prognosis and immunotherapy efficacy. In myeloid cell‐enriched hepatocellular carcinoma (HCC), elevated levels of TANs infiltration correlate strongly with unfavourable clinical outcomes.^18^ In non‐small‐cell lung cancer (NSCLC), neutrophils constitute the predominant immune population within the TME, with their proportion negatively correlated with CD4^+^/CD8^+^T cell infiltration.^22^, ^23^ Tumours with high stromal neutrophil infiltration exhibit an ‘immune‐cold’ phenotype, characterized by low T cell density and attenuated interferon‐γ (IFN‐γ) signalling.^24^, ^25^ The intratumoral neutrophil‐to‐CD8^+^T cell ratio has emerged as a robust indicator of ICI efficacy in NSCLC.^22^, ^23^ Similarly, in triple‐negative breast cancer (TNBC) and pancreatic ductal adenocarcinoma (PDAC), the accumulation of immunosuppressive neutrophils is significantly associated with sparse T cell infiltration and ICI resistance.^26^, ^27^

Elevated peripheral blood NLR and tissue TAN abundance emphasize the contribution of neutrophils to immune evasion in tumours. These clinical evidences not only provide potential biomarkers for predicting ICI efficacy but also drive mechanistic investigations into TAN functional regulation. Deciphering the multifaceted roles of neutrophils may thus represent a critical breakthrough in overcoming ICI resistance and optimizing combination therapy regimens.

### 1.2 TANs and ICI resistance

**FIGURE 2:**
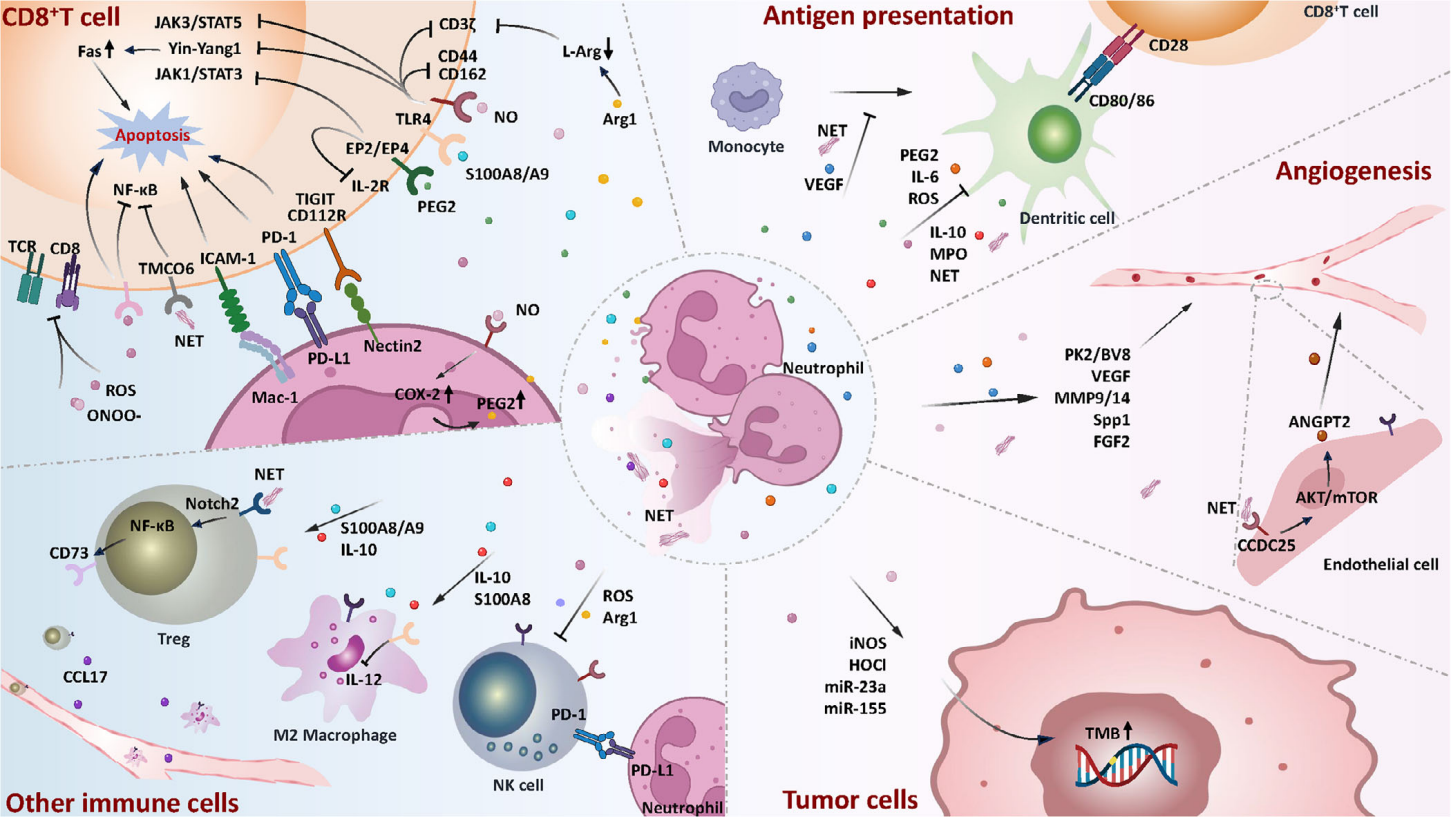
Neutrophils mediate resistance to immune checkpoint inhibitors (ICIs) through multiple mechanisms, (1) direct inhibition of cytotoxic T cell, including secretion of inhibitory factors (e.g., Arg1, PGE2, ROS, NO), contact‐mediated supression via immune checkpoint molecules (e.g., PD‐L1, Mac‐1), as well as neutrophil extracellular traps (NETs); (2) impairment of dendritic cell (DC) differentiation, maturation, and antigen presentation capacity; (3) activation of regulatory T cells (Tregs) and M2 macrophages and inhibition of natural killer (NK) cells; (4) remodel the microvascular network by secreting pro‐angiogenic factors (e.g., PK2/BV8, VEGF, FGF2); and (5) increase tumour mutation burden (TMB) through pro‐inflammatory mediators (e.g., iNOS, HOCl).

The predictive value of neutrophil‐related clinical indicators for ICI response highlights the crucial role of neutrophils in ICI resistance. In this section, we summarize the mechanisms by which TANs contribute to ICI resistance across multiple dimensions (Figure 2).

### 1.3 Inhibition of CD8^+^T cells

The therapeutic success of ICIs is largely contingent upon the activation of effector T cells, while TANs establish a T‐cell suppressive network through soluble factor secretion, cell‐contact‐dependent inhibition, and NET formation.

### 1.4 Soluble factor‐mediated suppression

#### 1.4.1 Arginase 1

Arginase 1 (Arg1), a key metabolic enzyme, is responsible for converting L‐arginine to ornithine and urea. As a primary contributor of Arg1 within the TME, neutrophils induce T cell dysfunction through L‐arginine depletion.^28^ Clinical samples demonstrate that peripheral blood Arg1^+^neutrophils increase with advancing tumour stages and exhibit a negative correlation with CD8^+^T cell proportions.^29^ Mechanistically, Arg1‐mediated L‐arginine deprivation downregulates CD3ζ chain expression on T cells, thereby inhibiting T cell proliferation, cytokine secretion and homing.^29^, ^30^, ^31^ In HNSCC tissues where TANs and T cells co‐localize, expression of granzyme B and Ki67 of T cells is strongly reduced, particularly those in close proximity to Arg1^+^neutrophils.^32^ These findings directly implicate TANs in suppressing effector T cell function via metabolic starvation.

#### 1.4.2 Prostaglandin E2

Neutrophil‐derived Prostaglandin E2 (PGE2) represents another critical mediator of T cell inhibition. Neutrophils exhibiting elevated fatty acid transport protein 2 (FATP2) levels exhibit increased arachidonic acid and PGE2 production.^33^ Secreted PGE2 induces T cell dysfunction through inhibition of the JAK1/STAT3 pathway and mitochondrial depolarization, thereby mediating resistance to immunotherapy.^33^, ^34^ Additionally, PGE2 binding to EP2/EP4 receptors on T cells leads to downregulation of IL‐2R expression, inhibiting IL‐2‐mediated CD8^+^T lymphocyte proliferation and attenuating antitumour responses.^35^, ^36^

#### 1.4.3 S100A8/9

Neutrophils represent the principal cellular origin of calprotectin S100A8/A9 in the TME.^37^ In melanoma patients, elevated serum S100A8/A9 concentrations may function as predictive biomarkers for anti‐PD‐1 treatment response.^38^, ^39^, ^40^ Among NSCLC patients receiving anti‐PD‐1 therapy, elevated blood levels of S100A8/A9 are markedly associated with lack of therapeutic response.^41^ Mechanistically, S100A8/A9 interacts with TLR4, leading to CD8^+^T cells depletion by inhibiting glycolysis, proliferation, and IFN‐γ production.^42^, ^43^

#### 1.4.4 Nitric oxide and reactive oxygen species

TANs also inhibit T cells’ function and drive ICI resistance through oxidative stress mediators, such as nitric oxide (NO) and reactive oxygen species (ROS). Neutrophils represent a major source of NO within the TME.^44^ TAN‐derived NO nitrosylates the CD3ζ chain of T cells, downregulating CD8 and chemokine receptor expression, thereby impairing T cell migration in response to chemokines.^45^ Concurrently, NO reduces E‐selectin expression on vascular endothelial cells and CD44/CD162 on T cells, disrupting T cell extravasation into tumour tissues.^46^, ^47^ At the level of T cell activation or apoptosis, NO disrupts JAK3/STAT5 phosphorylation by inhibiting guanylate cyclase activation in T cells, resulting in suppression of them.^48^ NO inactivates the transcriptional repressor Yin‐Yang1, resulting in upregulated Fas expression and enhanced susceptibility to Fas‐induced apoptosis in T cells.^46^, ^49^ Notably, NO may activate cyclooxygenase‐2 (COX‐2), enhancing PGE2 production and promoting release of Arg1, IL‐10, and VEGF by neutrophils—establishing a self‐reinforcing loop of metabolic inhibition that strengthens T cell suppression and ICI resistance.^50^

Neutrophil‐derived superoxide rapidly reacts with molecules such as H_2_O_2_, hydroxyl radicals, hypochlorous acid, and peroxynitrite to generate ROS. ROS exacerbate inflammation and stimulate apoptosis through damaging proteins, lipids, and DNA. Neutrophil‐derived ROS and peroxynitrite (products of ROS‐NO reactions) modify TCR and CD8 molecules, disrupting their binding to peptide‐MHC (pMHC) complexes and promoting antigen‐specific unresponsiveness in peripheral CD8^+^T cells.^51^ Additionally, ROS can also suppress activation of T cells through blocking the NF‐κB pathway and triggering apoptosis.^52^

### 1.5 Contact‐dependent inhibition

Beyond releasing immunosuppressive factors, TANs directly suppress CD8^+^T cells through contact‐dependent interactions. In ovarian cancer (OC), neutrophils activated by patient ascites supernatant induce T cell membrane endocytosis via Mac‐1(CD11b/CD18)‐mediated contact.^53^, ^54^ These structural and signalling alterations lead to T cell immunoparalysis via Mac‐1‐mediated intercellular contact, characterized by deficient nuclear factor of activated T cells (NFAT) translocation, reduced IL‐2 synthesis., and dysfunction in metabolic pathways including glucose uptake, mitochondrial activity, and mTOR signalling.^53^, ^54^ Neutrophil surface Mac‐1 engages intercellular adhesion molecule‐1 (ICAM‐1) on T cells to induce contact‐dependent T cell apoptosis.^55^, ^56^ However, blocking ICAM‐1 does not fully abrogate neutrophil‐mediated T cell inhibition, suggesting the involvement of additional T cell ligands.^56^

Similar to malignant cells, TANs exhibit PD‐L1 expression. Clinical investigations in NSCLC patients reveal that high frequencies of circulating PD‐L1^+^Arg1^+^ neutrophils are related to adverse prognosis.^57^ In glioma mouse models, TANs abundance correlates positively with PD‐L1 expression. T cell hyporesponsiveness to tumour antigens is inversely correlated with intratumoral neutrophil PD‐L1 levels.^58^ TANs directly induce T cell apoptosis, functional exhaustion, and suppression of IFN‐γ signalling through PD‐1/PD‐L1 interactions, thereby contributing to ICI resistance.^58^, ^59^, ^60^, ^61^, ^62^

Functioning as an immune checkpoint regulator, the cell adhesion molecule Nectin2 mediates TAN‐T cell crosstalk. Nectin2 on TANs suppresses T cell cytotoxicity through interactions with CD112R and T cell immunoglobulin and ITIM domain (TIGIT) on T cells.^63^, ^64^, ^65^, ^66^

### 1.6 Neutrophil extracellular traps

The reticular structure of NETs physically encapsulates tumour cells and impedes immune cell‐tumour cell interactions, directly shielding tumour cells from CD8^+^T cell cytotoxicity.^67^, ^68^ Immunosuppressive components released by NETs, such as IL‐10 and PD‐L1, suppress T cell activation and proliferation, inducing T cell dysfunction and dampening antitumour immune responses.^69^, ^70^ Emerging evidence shows that NET‐DNA binds to the transmembrane and coiled‐coil domain 6 (TMCO6) on T cells, inhibiting TCR signalling and nuclear translocation of NF‐κB, thereby blocking T cell activation.^71^ This extracellular matrix‐level inhibition synergizes with soluble factor‐mediated and contact‐dependent immunosuppression, collectively undermining the core effector pathways of ICIs.

### 1.7 Impairment of antigen‐presenting cells

Effective T cell activation relies on dual signalling: antigen recognition mediated by the TCR and co‐stimulatory signals from the engagement of CD28 on T cells with CD80/CD86 on antigen‐presenting cells (APCs).^72^ Dendritic cells (DCs) are central professional APCs. Impaired cross‐presentation by DCs profoundly hinders antitumour immunity, representing a critical mechanism of ICI therapy failure.

Neutrophils disrupt DC function through multifaceted mechanisms, including altered differentiation, impaired antigen presentation, and reduced survival, mediated by their secreted immunosuppressive factors.^73^ Vascular endothelial growth factor (VEGF), highly expressed in TANs, potently inhibits myeloid progenitor differentiation into DCs and attenuates antigen‐presenting capacity of DCs.^74^, ^75^ TAN‐derived PGE2 and IL‐6 reprogram classical DCs (cDCs) into immunosuppressive CD14^+^cDCs.^76^, ^77^, ^78^ Additionally, neutrophil‐released myeloperoxidase (MPO) induces lipid peroxidation, generating truncated lipid metabolites that directly inhibit DCs.^79^ Neutrophil‐derived ROS exert dual effects on DCs: physiological ROS levels enhance immune recognition by mediating DNA oxidation, whereas excessive ROS impairs phagosome acidification (due to proton depletion and increased pH) via nicotinamide adenine dinucleotide phosphate (NADPH) oxidase hyperactivation, impairing phagosomal acidification (proton depletion elevates pH) and reducing antigen presentation efficiency, thereby reducing antigen presentation efficiency.^80^, ^81^, ^82^

NETs also exhibit bidirectional effects on DCs. Early co‐culture of DCs with NETs upregulates costimulatory molecules CD80/CD86, enhancing antigen‐presenting capacity.^83^ Prolonged NETs exposure leads to caspase‐dependent and apoptosis‐inducing factor‐mediated DC apoptosis, driven by NETs’ histone and elastase.^83^ Additionally, NETs downregulate IL‐4 receptor expression in monocytes, blocking their differentiation into mature DCs, while neutrophil elastase (NE) induces regulatory T cells' (Tregs) generation when applied to immature DCs in vitro.^84^, ^85^ Neutrophils and NETs contribute to IL‐10 within the TME. Excessive IL‐10 signalling impairs IL‐12 secretion and abolishes cross‐presentation capacity in tumour‐infiltrating CD103^+^cDC1 subsets, ultimately leading to T cell dysfunction.^86^

### 1.8 Modulation of other immune cells

Beyond effector T cells, TANs significantly influence the functions of Tregs, TAMs, and NK cells through complex cell‐cell interactions, further establishing an immunosuppressive microenvironment that promotes ICI resistance.

#### 1.8.1 Tregs

Tregs highly express CTLA‐4, engaging CD80/CD86 on DCs, to impair APC‐dependent T cell activation and enhance their immunosuppressive properties.^87^ Promotion of Tregs represents a critical pathway for neutrophil‐mediated T cell suppression and ICI resistance.^88^, ^89^ In RET‐transgenic melanoma models, infiltration of CCR5^+^Arg1^+^PD‐L1^+^TANs correlates with increased Treg proportions in tumours.^90^ In bladder cancer patients, neutrophil‐secreted CCL17 recruits Tregs to tumour foci in a concentration‐dependent manner.^91^, ^92^ Cytokine IL‐10 mediates the formation of a reciprocal activation loop between TANs and Tregs—TAN‐derived IL‐10 promotes Tregs activation, which in turn enhances the immunosuppressive phenotype of neutrophils.^93^ Neutrophil‐derived S100A8/A9, acting as damage‐associated molecular patterns, also promote positive feedback.^94^ NETs can modulate the differentiation and function of Tregs. NETs upregulate CD73 on Tregs expression via notch2‐mediated NF‐κB activation, promoting their infiltration into the TME and reducing ICI efficacy.^95^ Concurrently, NETs drive Treg differentiation through toll‐like receptor 4 (TLR4)‐mediated modulation of mitochondrial oxidative phosphorylation in naïve CD4^+^ T cells.^96^

#### 1.8.2 TAMs

Neutrophils influence macrophage recruitment and polarization through cytokine secretion. TANs frequently secrete chemokines CCL2 and CCL17, which enhance macrophage migratory activity.^97^ TAN‐secreted S100A8 binds to TLR4 on TAMs, mediating immunosuppression though PI3K‐AKT, STAT3, and NF‐κB pathways activation and elevating *PD‐L1* transcription.^98^ TAN‐derived IL‐10 drives macrophage polarization towards the immunosuppressive M2 phenotype. IL‐10 reduces macrophage IL‐12 secretion, reprogramming them from antitumour M1 to pro‐tumour M2 states. This TAN‐TAM crosstalk creates an immune‐suppressive microenvironment that facilitates tumour escape, concurrently dampening antitumour immune responses and compromising ICI efficacy.^99^

#### 1.8.3 NK cells

Neutrophils suppress NK cell activation, resulting in decreased NK cell cytotoxicity in tumours.^100^, ^101^ In colorectal cancer (CRC) models, neutrophils hinder NK cell infiltration by suppressing CCR1 expression on NK cells and impair their antitumour functions via PD‐L1/PD‐1 axis‐mediated interactions.^102^ Cathepsin released from NETs specifically cleaves the NK cell activating receptor NKp46, suppressing IFN‐γ production and degranulation to attenuate cytotoxicity.^103^ Furthermore, neutrophil‐derived immunosuppressive factors such as Arg1 and ROS inhibit NK cell anti‐tumour activity by depleting microenvironmental nutrients or inducing oxidative stress.^104^ Impaired NK cell activity not only reduces their direct killing of tumour cells but also weakens their synergistic antitumour effects with CD8^+^ T cells, ultimately compromising the efficacy of ICIs.

### 1.9 Promotion of angiogenesis

Neovascularization promotes disease progression and ICI resistance by impeding T cell infiltration, impairing T cell function, and inducing hypoxia‐driven expression of immunosuppressive molecules.^105^, ^106^ TANs represent a principal origin of pro‐angiogenic growth factors in the TME.^107^, ^108^, ^109^ Their highly expressed prokineticin 2 (PK2/BV8), a ligand for G protein‐coupled receptors PKR1/2, directly enhances endothelial cell proliferation and lumen formation by activating MAPK and PI3K pathways.^110^, ^111^ Stimulated by granulocyte‐macrophage colony‐stimulating factor (GM‐CSF), neutrophils secrete oncostatin M (OSM), subsequently inducing VEGF production in tumour cells via the JAK‐STAT pathway.^112^ TAN‐derived Spp1 (osteopontin/OPN) and MMP14 facilitate neovascularization by regulating endothelial cell chemotaxis and branching morphogenesis.^113^ Additionally, fibroblast growth factor 2 (FGF2) from TANs can exert compensatory pro‐angiogenic effects after VEGF pathway blockade.^114^

NETs contribute to angiogenesis through multiple pathways. Neutrophil elastase (NE) released by NETs proteolytically liberates pro‐angiogenic mediators like VEGF‐ or platelet‐derived growth factor (PDGF). Meanwhile, NET‐DNA binds to coiled‐coil domain containing 25 (CCDC25) on endothelial cells, triggering AKT/mTOR activation that enhances angiopoietin‐2 (ANGPT2) synthesis and directly accelerates endothelial proliferation and angiogenesis.^115^ These mechanisms collectively highlight TANs as critical architects of the pro‐angiogenic, immune‐suppressive TME.

### 1.10 Increase of tumour mutation burden

Tumour mutation burden (TMB), reflecting the overall mutational load of the tumour genome, not only drives malignant progression but also correlates with ICI resistance. Elevated TMB in patients undergoing ICI treatment correlates with reduced response rates and diminished clinical benefit across various malignancies.^116^ The regulation of TMB involves diverse mechanisms, such as the inactivation of tumour suppressor genes and the stimulation of oncogenic signalling. For example, deletion of the tumour suppressor *LKB1/STK11* promotes the accumulation of pro‐tumour TANs and confers resistance to ICIs in lung cancer,^117^, ^118^ while loss of *PTEN* drives excessive PI3K‐AKT signalling activation, inducing expression of immunosuppressive cytokines that enhance TAN accumulation and reduce T cell recruitment in tumours.^119^ In this process, TANs‐released nitric oxide synthase (iNOS) and hypochlorous acid (HOCl) can cause DNA damage and mutations in the *HPRT* gene of premalignant epithelial cells.^120^, ^121^ In addition, neutrophils release pro‐inflammatory miR‐23a and miR‐155, compromising DNA repair mechanisms and elevating genomic instability.^122^ Notably, elevated TMB and TAN infiltration exhibit a synergistic effect: while high‐mutation tumour cells may generate more neoantigens, they also promote TAN recruitment through aberrant signalling, forming a ‘high mutation burden‐strong immune suppression’ resistant phenotype.

### 1.11 Targeting neutrophils in combination with ICI therapy

**FIGURE 3:**
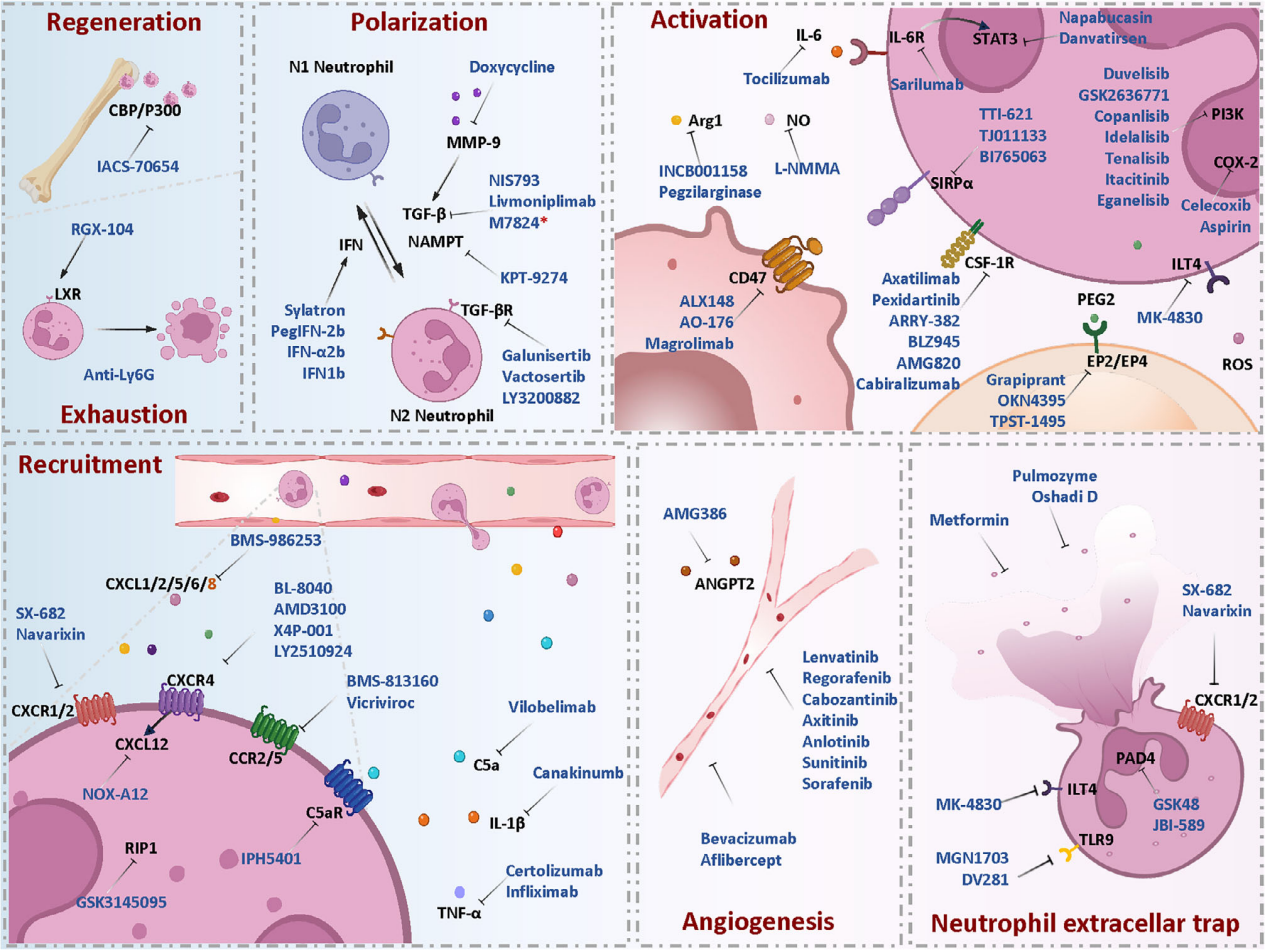
Multidimensional neutrophil‐targeted therapies combination with ICIs: (1) Targeted inhibition of CBP and activation of LXR synergize with ICIs by suppressing neutrophil production and inducing neutrophil depletion, respectively. (2) Targeted drugs against chemokine receptors CXCR1/2/4, CCR2/5 and chemokines C5a, IL‐1β, and TNF‐α in combination with ICIs enhance the efficacy of ICIs by inhibiting neutrophil recruitment. (3) Targeted inhibition of the TGF‐β/TGF‐βR axis, NAMPT, MMP‐9 or application of IFN drugs induces neutrophils to polarize towards the anti‐tumour N1 phenotype. (4) Targeted inhibition of the IL‐6/IL‐6R/STAT3 axis, CD47/SIRPα axis, GM‐CSF/CSFR axis, PI3K signalling pathway and inhibitory factors (e.g., Arg1, PGE2, ROS, NO) suppresses neutrophil activation. (5) Anti‐angiogenic drugs in combination with ICIs alleviate neutrophil‐induced angiogenesis. (6) Targeted inhibition of NETs production or promotion of NETs digestion synergizes with ICI treatment.

Targeted intervention strategies against the complex resistance mechanisms mediated by TANs are shifting from single effector molecule blockade to multidimensional network regulation. In recent years, drug development has focused on pathways involved in TAN recruitment/activation, functional reprogramming, and NETs formation. The combined application of drugs targeting neutrophils and ICIs has demonstrated synergistic antitumour effects, providing new pathways to overcome resistance bottlenecks. In this section, we systematically elaborate on core therapeutic strategies by integrating mechanistic insights and clinical translation potential (Figure 3).

### 1.12 Targeting neutrophil exhaustion and regeneration

#### 1.12.1 Neutrophil exhaustion

Drugs targeting specific antigens on neutrophils can reshape the immune microenvironment by eliminating pathogenic TANs. Neutrophil depletion mediated by anti‐Ly6G antibodies notably inhibits tumour progression and improves therapeutic outcomes through enhancing cytotoxic T cell infiltration and decreasing Treg recruitment.^123^, ^124^ Exhaustion of CD8^+^T cells or NK cells completely abolishes the efficacy of Ly6G inhibitors, confirming that their antitumour effects rely primarily on relieving suppression of adaptive immunity. Preclinical studies show that combination therapy with anti‐Ly6G antibodies and PD‐L1 inhibitor enhances cytotoxic T cells infiltration, significantly reducing tumour burden and improving the efficacy of ICB,^123^, ^124^ although no Ly6G‐targeting drug has entered clinical trials to date. Liver X receptor (LXR) agonist RGX‐104 inhibits neutrophil survival by activating the apolipoprotein E (ApoE) signalling pathway and reduces neutrophil production via regulating the function of the hematopoietic niche.^125^, ^126^ A phase I trial demonstrated that RGX‐104 decreases circulating neutrophil proportion in CRC and renal cell carcinoma (RCC), thus enhancing sensitivity to PD‐1 blockade (NCT02922764).^126^

#### 1.12.2 Neutrophil regeneration

Beyond depletion strategies, modulating neutrophil generation represents a potential therapeutic direction. During tumour growth, pathogen‐associated molecular patterns (PAMPs) and inflammatory signals drive abnormal differentiation of myeloid progenitors into neutrophils by activating TLR receptors on hematopoietic stem and progenitor cells.^127^ The CBP/P300 bromodomain inhibitor IACS‐70654 impedes tumour‐induced aberrant neutrophil progenitor differentiation through suppression of IL‐3, thereby decreasing TAN numbers and significantly enhancing ICI response.^128^ These findings suggest that targeting neutrophil generation and maintenance may optimize the immunomodulatory effects of ICIs.

### 1.13 Targeting neutrophil recruitment

Peripheral neutrophils are mobilized to the TME by various cytokines and chemokines, establishing a defensive barrier that impedes CD8^+^T cells’ penetration, and producing immunosuppressive mediators to induce immune escape. Given the potential side effects of neutrophil depletion, combining ICIs with agents targeting neutrophil recruitment‐related cytokines represents a more optimized strategy to enhance anti‐tumour immunity (Table 1; Table S2).

**TABLE 1:** Clinical trials of ICIs combined with drugs targeting neutrophil recruitment.

Target	Drug	ICIs	Trial number	Cancer type	Group	ORR	DCR	Median PFS (month)	Median OS (month)
CXCR1/2	Navarixin	Pembrolizumab	NCT03473925	NSCLC/Castration‐resistant PC/MSS CRC	Total	2.9% (3/105)	‐	1.8‐2.4	6.5–13.0
					CRPC	5% (2/40)	‐	2.1	10.8–11.2
					MSS CRC	2.5% (1/40)	‐	1.8–1.9	6.5–8.0
					NSCLC	0% (0/25)	‐	2.1–2.4	12.0–13.0
CXCR4	BL‐8040/BKT140	Pembrolizumab	NCT02907099	M/R/Stage IV PDAC	‐	6.7% (1/15)	‐	‐	‐
			NCT02826486	M PDAC	BL‐8040 + Pembrolizumab	3.3% (1/30)	33.3% (10/30)	1.5	3.3
					BL‐8040 + Pembrolizumab + Chemotherapy	20.5% (8/39)	64.1% (25/39)	3.8	6.6
	Mavorixafor/X4P‐001	Nivolumab	NCT02923531	ccRCC	‐	‐	55.5% (5/9)	‐	‐
	LY2510924	Durvalumab	NCT02737072	Solid tumours	‐	0% (0/9)	44.4% (4/9)		
CCR2/5	BMS‐813160	Nivolumab	NCT03496662	Borderline resectable/Locally advanced PDAC	Borderline resectable PADC	42% (7/17)	‐	11.9	18.2
					Locally advanced PADC	20% (3/15)	‐	14.7	17
CCR5	Vicriviroc	Pembrolizumab	NCT03631407	Advanced MSS CRC	‐	5% (2/40)	‐	2.1	4.6–5.3
CCR4	Mogamulizumab	Nivolumab	NCT02705105	Solid tumour	Squamous cell NSCLC	20% (1/5)	‐	‐	‐
					PD‐L1^−^nonsquamous NSCLC	25% (1/4)	‐	‐	‐
					HNSCC	10% (1/10)	‐	‐	‐
					Non‐MSI High CRC	3.4% (1/29)	‐	‐	‐
					OC/FTC/PPC	14.3% (3/21)	‐	‐	‐
					HCC	16.7% (4/24)	‐	‐	‐
					PDAC	0% (0/17)	‐	‐	‐
C5a	Vilobelimab	Pembrolizumab	NCT04812535	SCC of skin	Vilobelima	10% (1/10)	‐	1.7	9.5
					Vilobelimab+ Pembrolizumab	20% (3/15)	‐	2.9–3.5	3.5–10
IL‐1β	Canakinumb	Pembrolizumab	NCT03968419	NSCLC	Canakinumb	0% (0/35)	‐	‐	‐
					Pembrolizumab	11.1% (2/18)	‐	‐	‐
					Canakinumb+ Pembrolizumab	8.6% (3/35)	‐	‐	‐
			NCT03631199	NSCLC	Canakinumab +Pembrolizumab +CTx	45.6% (146/320)	86.9% (278/320)	6.77	20.83
					Placebo +Pembrolizumab+CTx	45.5% (147/323)	84.8% (274/323)	6.77	20.17
TNF‐α	Certolizumab/ Infliximab	Nivolumab+ Ipilimumab	NCT03293784	Melanoma	Infliximab	50% (3/6)	‐	‐	‐
					Certolizumab	100% (7/7)	‐	‐	‐
RIP‐1	GSK3145095	Pembrolizumab	NCT03681951	Advanced solid tumours	‐	0% (0/8)	‐	‐	‐

Abbreviations: CRC, colorectal cancer; CRPC, castration‐resistant prostate cancer; CTx, platinum‐based doublet chemotherapy; DCR, disease control rate; FTC, fallopian tube cancer; HCC, hepatocellular carcinoma; MSS, microsatellite stable; NSCLC, non‐small‐cell carcinoma; OC, ovarian cancer; ORR, objective response rate; OS, overall survival; PADC, pancreatic ductal adenocarcinoma; PFS, progression‐free survival; PPC, primary peritoneal carcinoma.

### 1.14 CXCR1/2 axis

C‐X‐C chemokines regulate neutrophil recruitment and activation by binding to CXCR1/2 on neutrophils.^129^, ^130^, ^131^ Preclinical studies show that CXCR1/2 inhibitors (SX‐682, navarixin) could reduce TANs accumulation, increase PD‐1^+^CD8^+^T cell abundance, and sensitize tumors to combined anti‐CTLA‐4/PD‐1 therapy.^26^, ^132^, ^133^, ^134^ Among them, navarixin combined with pembrolizumab has entered the clinical trial (NCT03473925) for previously treated advanced solid tumours.^135^ Though prematurely halted owing to insufficient efficacy in the interim analysis, its manageable safety profile still provides critical insights for further development.^135^ Currently, clinical trials of SX‐682 combined with pembrolizumab or nivolumab are actively recruiting participants for melanoma (NCT03161431), recurrent/metastatic lung cancer (NCT05570825), CRC (NCT04599140), and pancreatic cancer (NCT04477343).

### 1.15 CXCL8/IL‐8 pathway

As the most potent neutrophil chemokine, elevated serum CXCL8/IL‐8 levels strongly predict poor outcomes in NSCLC or advanced melanoma patients receiving ICI therapies.^136^ The anti‐IL‐8 antibody BMS‐986253 reduces TAN recruitment and promotes T cell infiltration by blocking CXCL8‐CXCR1/2 interactions. Clinical trials of this agent combined with nivolumab are ongoing for advanced HCC (NCT04050462), NSCLC (NCT04123379), prostate cancer (NCT03689699), and melanoma (NCT04572451, NCT03400332).

### 1.16 CXCR4/CXCL12 axis

CXCR4‐mediated neutrophil recruitment plays a critical role in pre‐metastatic niche formation.^137^ CXCR4 inhibitors (BL‐8040/BKT140, mavorixafor/X4P‐001, LY2510924) reduce TAN infiltration by blocking CXCL12 signalling. Although the objective response rate (ORR) of BL‐8040 plus pembrolizumab was only 3.3% in the phase II trial (NCT02826486) for PDAC, patients exhibited enhanced T‐cell function. Notably, the triple therapy consisting of BL‐8040, pembrolizumab, and chemotherapy achieved an ORR of 20.5%.^138^, ^139^ A phase I study of X4P‐001 plus pembrolizumab in melanoma (NCT02823405) showed increased CD8^+^T cell infiltration, antigen presentation, and IFN‐γ expression with acceptable safety.^140^ In a clinical trial evaluating X4P‐001 plus nivolumab for metastatic clear cell RCC patients unresponsive to ICI monotherapy (NCT02923531), 4 of 9 patients with disease progression achieved stabilization, and 1 of 5 stable patients achieved partial response, demonstrating the potential antitumour efficacy of CXCR4‐targeting drugs plus ICIs.^141^ Additionally, NOX‐A12, a CXCL12 inhibitor, combined with pembrolizumab in pretreated metastatic PDAC/CRC, demonstrated good tolerability and provided direct evidence of T cell migration and accumulation in responsive tissues (NCT03168139).^142^

### 1.17 Other chemotactic pathways

The CCR5 pathway (ligands CCL3/4/5) drives neutrophil recruitment in melanoma and NSCLC.^57^ However, CCR5‐targeting treatment combined with ICIs has yielded differential efficacy across different tumour types. The CCR5 inhibitor vicriviroc plus pembrolizumab was evaluated in CRC (NCT03631407), yielding only a 5% ORR.^143^ In contrast, the CCR2/5 inhibitor BMS‐813160 combined with nivolumab and chemotherapy (NCT03496662) achieved ORRs of 42% and 20% in borderline resectable or locally advanced PDAC, indicating potential efficacy against ‘cold tumours’.^144^

Beyond classical chemotactic pathways, non‐classical neutrophil recruitment pathways, such as the complement C5a‐C5aR axis, also contribute to neutrophil recruitment.^145^, ^146^ A phase II trial (NCT04812535) testing the anti‐C5a antibody vilobelimab combined with pembrolizumab demonstrated a 33.3% ORR in cutaneous squamous cell carcinoma, demonstrating promising reactivity. Vilobelimab has been approved by the FDA for emergency use in hospitalized adults due to its anti‐inflammatory effects in severe infections, suggesting its potential ability in mitigating ICI treatment‐induced inflammatory side effects.^147^

### 1.18 Inflammatory signalling pathway

Neutrophils can also enhance tumour infiltration and immunosuppression via IL‐17‐dependent manners.^148^ While inhibition of IL‐6 or GM‐CSF signalling might disrupt IL‐17A‐associated TAN infiltration, no combination therapies targeting IL‐17 have entered clinical trials.^149^, ^150^ IL‐1β triggers γδT cells to generate IL‐17, leading to G‐CSF‐dependent neutrophil expansion and polarization.^151^ Although combining anti‐IL‐1β or anti‐IL‐1R therapies with ICIs may enhance anti‐tumour immunity,^152^ the phase III trial of the IL‐1β inhibitor canakinumab plus pembrolizumab for first‐line advanced/metastatic NSCLC failed to show a significant survival benefit (NCT03631199),^153^ highlighting the need for validation in other tumour types.

Tumour necrosis factor (TNF) signalling can reduce the ICI response by participating in TAN recruitment and directly affecting T cell function. In PDAC mouse models, inhibiting the TNF/RIP1/RIP3 pathway decreases TAN infiltration.^154^ Preclinical studies in melanoma showed that TNF inhibition attenuated T cell apoptosis and suppressed PD‐L1/TIM‐3 upregulation induced by anti‐PD‐1 therapy.^155^ In a clinical trial of TNF‐α inhibitors (certolizumab, infliximab) combined with ICIs for melanoma, the ORR reached 100% in 7 patients receiving certolizumab combination therapy and 50% in 6 patients treated with infliximab combination therapy (NCT03293784).^156^ Signs of systemic T cell activation and maturation were observed, indicating the promising potential of TNF‐blocking therapy combined with ICIs.^156^ However, validation of efficacy necessitates further investigation in larger patient populations. Receptor‐interacting proteins (RIPs), critical for cell death and inflammation, influence neutrophil chemotaxis and recruitment by regulating chemokine release and inflammatory signalling. Several RIP‐1 inhibitors are currently used for treating inflammatory diseases.^157^ The evaluation of the RIP1 kinase inhibitor GSK3145095 plus pembrolizumab (NCT03681951) in PDAC patients failed to demonstrate a survival‐prolonging effect.^158^ The role of this combination in melanoma is still under evaluation (NCT05034536).

### 1.19 Emerging targets

Numerous other neutrophil recruitment‐related molecules contribute to ICI resistance. In HCC, upregulation of the CRKL/β‐catenin/VEGFα axis mediates PD‐1 inhibitor resistance by enhancing TANs infiltration, supporting the strategy of combining CRKL inhibitors with anti‐PD‐1 agents.^159^ ACSL6‐regulated CXCL1/5 expression facilitates TAN recruitment, and its deletion or mutation enhances cytotoxic cell activity and the PD‐1 inhibitor efficacy.^160^ Additionally, sialic acid‐modified colchicine derivatives specifically block TAN infiltration into tumours, reducing their suppressive effects on T cells and significantly enhancing the efficacy of PD‐L1 antibodies in advanced tumour models.

These studies collectively stress the broad promise of strategies targeting neutrophil recruitment in combination with ICB. However, as these findings are currently limited to preclinical research, further clinical studies are essential to assess the safety and effectiveness of such combinations.

### 1.20 Targeting neutrophil polarization phenotypes

#### 1.20.1 IFN/TGF‐β signal

The functional heterogeneity of TANs arises from microenvironment‐driven polarization, where the dynamic balance between type I interferon (IFN) and transforming growth factor‐β (TGF‐β) signalling serves as a hub regulating the conversion between N1 and N2 phenotypes. Interventional strategies targeting polarization aim to reverse ICI resistance by reshaping TAN functional states, which has become a critical direction in tumour therapy (Table 2; Table S3).^161^ In the IFN‐related strategies, six trials have explored combinations of ICIs with type I IFNs (sylatron, pegIFN‐α2b, IFN‐α2b, IFN1b). IFN‐α2b combined with ipilimumab (anti‐CTLA‐4) demonstrated objective efficacy signals in advanced melanoma, achieving a pathological complete response rate of 32% and a significant increase in tumour‐infiltrating T cells (NCT01496807, NCT01608594).^162^, ^163^ A clinical trial of sylatron in combination with ipilimumab in melanoma demonstrated favourable clinical efficacy, with an ORR of 40% (NCT01496807). PegIFN‐α2b plus pembrolizumab showed limited antitumour activity in phase Ib trials for advanced melanoma or RCC, with response rates of 20% and 17%, respectively (NCT02089685).^164^ Eight trials evaluate TGF‐β receptor (TGF‐βR) inhibitors (galunisertib/LY2157299, LY3200882, vactosertib/TEW‐7197) in combination with ICIs. Among these, galunisertib plus nivolumab achieved a 24% ORR and a disease control rate (DCR) of 40% in relapsed NSCLC (NCT02423343).^165^ Three trials evaluating TGF‐β antibodies (NIS793, livmoniplimab) plus ICIs have focused on blocking the paracrine TGF‐β effects to relieve dual suppression of neutrophils and T cells.

**TABLE 2:** Clinical trials of ICIs combined with drugs targeting neutrophil polarization.

Target	Drug	ICIs	Trial number	Cancer type	Group	ORR	DCR	Median PFS (month)	Median OS (month)
IFN	Sylatron		Ipilimumab	NCT01496807	Melanoma		40% (12/30)	‐	5.9
			Pembrolizumab	NCT02982720	Advanced Cholangiocarcinoma		0% (0/4)	‐	‐
TGF‐β	M7824/Bintrafusp alfa		NCT03427411	HPV‐associated malignancies	Total	30.5% (18/59)	44.1% (26/59)	1.4–3.5	4.4–19.2
+PD‐L1					ICB‐naive	‐	62.1% (18/29)	3.5	19.2
					ICB‐resistant	‐	19.2% (5/26)	1.4	4.4
			NCT04287868	Advanced HPV‐associated malignancies	Total	22% (11/50)	‐	‐	‐
					HPV16^+^tumors	29.7% (11/37)	‐	‐	‐
					HPV16^+^tumors, ICB‐naive	62.5% (5/8)	‐	‐	‐
					HPV16^+^tumors, ICB‐resistant	20.7% (6/29)		‐	‐
			NCT03833661	Locally advanced or metastatic BTC	‐	10.7% (17/159)	‐	1.8	7.6
			NCT04066491	BTC	M7824+ GEM+ DDP	31.5% (23/73)	61.6% (45/73)	5.5	11.5
					Placebo+ GEM+ DDP	19.5% (15/77)	76.6% (59/77)	5.6	11.5
			NCT04246489	UC	‐	21.9% (32/146)	‐	1.9	13.7
			NCT03451773	Previously treated advanced PDAC	‐	0% (0/7)	57.1% (4/7)	1.4	3.5
			NCT03554473	Relapsed SCLC	M7824	10% (1/10)	‐	2.31	4.58
					M7824+TPT	0% (0/5)	‐	1.44	2.89
					M7824+TMZ	20% (3/15)	‐	2.03	8.86
			NCT03436563	Metastatic CRC/advanced solid tumours with MSI	‐	‐	‐	2‐year PFS 75% (3/4)	2‐year OS 100% (4/4)
			NCT02517398	Solid tumours	Dose‐escalation	10.5% (4/38)	‐	1.5	13.8
					Dose‐expansion	8.8% (6/68)	‐	1.4	13.5
			NCT02699515	Solid tumours	BTC	20% (6/30)	40% (12/30)	2.5	12.7
					GC	19.4% (6/31)	22.6% (7/31)	1.3	10.1
					ESCC	10% (3/30)	20% (6/30)	1.4	11.9
			NCT03631706	Unresectable NSCLC	M7824	‐	‐	7	21.1
					Pembrolizumab	‐	‐	11.1	22.1
			NCT03840915	Stage IV NSCLC	M7824+DDP/CBP+Pemetrexed	45% (18/40)	‐	5	11.4
					M7824+CBP+PTX	66.7% (6/9)	‐	4.1	11.8
					M7824+DDP/CBP+GEM	44.4% (4/9)	‐	5.4	‐
					M7824+DOC	16.7% (2/12)	‐	2.6	16.5
			NCT04247282	Resectable HNSCC not associated with HPV infection	‐	‐	‐	1‐year PFS 86% (12/14)	‐
			NCT04220775	Recurrent or second primary HNSCC	M7824+SBRT	50% (1/2)	‐	‐	‐
			NCT04349280	Metastatic or locally advanced UCC	‐	20% (5/25)	‐	‐	‐
TGF‐βR	Galunisertib/LY2157299	Nivolumab	NCT02423343	Advanced refractory solid tumours	NSCLC	24% (6/25)	40% (10/25)	5.26	11.99
MMP	Doxycycline	Ipilimumab	NCT01590082	Melanoma		0% (0/10)	‐	‐	‐
NAMPT	KPT‐9274	Nivolumab	NCT02702492	Solid tumours		0% (0/60)	‐	‐	‐

Abbreviations: ORR, objective response rate; DCR, disease control rate; PFS, progression‐free survival; OS, overall survival; NSCLC, non‐small‐cell carcinoma; SCLC, small‐cell carcinoma; PADC, pancreatic ductal adenocarcinomas; BTC, biliary tract cancer; GC, gastric cancer; ESCC, esophageal squamous cell carcinoma; HNSCC, head and neck squamous cell carcinoma; UC, urothelial cancer; HPV, human papillomavirus; ICB, immune checkpoint blockade; GEM, gemcitabine; DDP, cisplatin; CBP, carboplatin; TX, paclitaxel; DOC, docetaxel; TPT, topotecan; TZM, temozolomide; SBRT, stereotactic body radiation therapy.

Notably, an additional 33 clinical trials evaluate the efficacy of the bispecific antibody bintrafusp alfa/M7824, targeting both TGF‐β and PD‐L1. Currently, M7824 have demonstrated effective activity and achieved enhanced response rates in multiple tumours.^166^, ^167^, ^168^ In advanced human papillomavirus (HPV)‐associated malignancies, phase I/II trials have reported an ORR of 30.5% (NCT02517398, NCT03427411), and a 21.9% ORR was observed in recurrent/metastatic cervical cancer patients within the trial NCT04246489.^169^, ^170^ The ORR in ICB‐naive HPV16^+^ tumours reached 62.5%. Even in ICB‐resistant HPV16^+^ tumours, M7824 still achieved an ORR of 20% (NCT04287868). In a trial for biliary tract cancer (BTC) patients progressing after first‐line chemotherapy, bintrafusp alfa showed a 20% ORR (NCT02699515).^171^ The regimen of M7824 plus gemcitabine and cisplatin achieved an ORR of 31.5% in BTC, significantly higher than the 19.5% ORR with the gemcitabine plus cisplatin chemotherapy regimen (NCT04066491). However, in locally advanced/metastatic chemotherapy‐refractory BTC, the ORR dropped to 10.7% (NCT03833661).^172^ A phase III trial in advanced NSCLC showed no superior progression‐free survival of bintrafusp alfa compared with pembrolizumab monotherapy (NCT03631706).^173^ This discrepancy may be attributed to the heterogeneous role of TGF‐β signalling across different tumour types and patient populations. In advanced NSCLC, particularly in unselected patients, the immunosuppressive TME may be driven by dominant pathways other than TGF‐β, or baseline levels of active TGF‐β might be insufficient for the drug to demonstrate a clear synergistic effect over PD‐L1 inhibition alone. This also highlights the importance of seeking biomarkers that can screen for TGF‐β‐driven immunosuppressive phenotypes. Furthermore, M7824 has also demonstrated varying degrees of efficacy and safety in tumours such as urothelial carcinoma (UC), HNSCC, and uterine cervical carcinoma (UCC). These agents simultaneously block immune checkpoint inhibition and TGF‐β‐induced N2 polarization, representing a ‘dual‐targeting + phenotypic reprogramming’ paradigm that synergizes innate and adaptive immunity.^161^

### 1.21 Other signals

For other polarization‐related targets, MMP‐9 inhibitors reverse TAN pro‐tumour functions by blocking TGF‐β activation, with doxycycline combined with ipilimumab tested in melanoma (NCT01590082).^174^ Innovative strategies include low‐dose endotoxin training, which activates the STAT5 pathway and reduces interleukin‐1 receptor‐associated kinase (IRAK‐M) expression in neutrophils, inducing a unique immune‐enhancing phenotype.^175^ Regulation of the endoplasmic reticulum stress pathway inositol‐requiring enzyme 1α (IRE1α)‐X‐box binding protein 1 (XBP1) has also emerged as a target.^176^ Genetic deletion of IRE1α in neutrophils delays tumour progression and enhances ICI efficacy by impairing N2 polarization.^176^ These preclinical findings require clinical validation to translate mechanistic insights into therapeutic benefits. Collectively, targeting neutrophil polarization addresses the phenotypic plasticity of TANs, offering opportunities to reprogram their functions from immunosuppressive to immunopotentiation.

### 1.22 Targeting neutrophil activation and release of immunosuppressive factors

The immunosuppressive function of TANs depends on their activation status and release of effector molecules. Targeting key signalling pathways and secreted factors can block inhibitory cascades, synergizing with ICIs to exert antitumour effects (Table 3; Table S4).

**TABLE 3:** Clinical trials of ICIs combined with drugs targeting neutrophil activation.

Target	Drug	ICI	Trial Number	Cancer Type	Group	ORR	DCR	Median PFS (month)	Median OS (month)
IL‐6	Tocilizumab	Nivolumab, Ipilimumab	NCT03999749	Melanoma	‐	48.6% (34/70)	‐	‐	‐
STAT3	BBI‐608/	Pembrolizumab	NCT02851004	Metastatic CRC	MSI‐H CRC	50% (5/10)	90% (9/10)	‐	‐
	Napabucasin				MSS CRC	10% (4/40)	45% (18/40)	1.6	7.3
GM‐CSF	Sargramostim	Ipilimumab	NCT01134614	Unresectable Stage III or IV melanoma	Ipilimumab	‐	‐	3.1	12.7
					Sargramostim +Ipilimumab	‐	‐	3.1	17.5
CSF‐1R	ARRY‐382	Pembrolizumab	NCT02880371	Advanced solid tumours	Phase 1b	10.5% (2/19)	‐	1.4–4.7	7.4–14.7
					Phase 2 PDAC	3.70%	‐	1.4	2.2
	Pexidartinib/	Pembrolizumab	NCT02452424	Advanced melanoma and other solid tumours	Melanoma	15.4% (2/13)	‐	‐	‐
	PLX3397				OC	6.7% (1/15)	‐	‐	‐
					GIST	0% (0/6)	‐	‐	‐
					NSCLC	0% (0/8)	‐	‐	‐
					HNSCC	0% (0/3)	‐	‐	‐
	BLZ945	Spartalizumab/PDR001	NCT02829723	Advanced solid tumours	BLZ945	BOR 13.6% (3/22)	27.3% (6/22)	‐	‐
					BLZ945+PDR001	BOR 0% (0/21)	23.8% (5/21)	‐	‐
	AMG820	Pembrolizumab	NCT02713529	Advanced solid tumours	Total	2.6% (3/116)	‐	2.1	5.3
					pMMR CRC	4.9% (2/41)	‐	‐	‐
					NSCLC	3.4% (1/29)	‐	‐	‐
					PDAC	0% (0/31)	‐	‐	‐
	Cabiralizumab/FPA008	Nivolumab	NCT02526017	Advanced solid tumours	NSCLC (PD‐1 naïve)	6.9% (/29)	‐	‐	‐
					NSCLC (PD‐1 resistant)	3.2% (1/31)	‐	‐	‐
					HNSCC	13.8% (4/29)	‐	‐	‐
					OC	13.3% (4/30)	‐	‐	‐
					Melanoma	9.1% (1/11)	‐	‐	‐
					RCC	6.7% (2/30)	‐	‐	‐
					PDAC	5.9% (4/68)	‐	‐	‐
					Malignant glioma	0% (0/30)	‐	‐	‐
			NCT03158272	Advanced malignancies	Cabiralizumab	‐	50% (2/4)	‐	‐
					Cabiralizumab +Nivolumab	‐	33.3% (3/9)	‐	‐
			NCT03697564	Stage IV PC	‐	‐	‐	6‐month PFS 50% (1/2)	6‐month OS 50% (1/2)
			NCT03599362	Locally advanced unresectable PC	Surgical resection rate:25% (1/4)				
			NCT03336216	Advanced PC	FPA008 +Nivolumab	4.44% (2/49)	‐	1.81	2.2
					FPA008 +Nivolumab +GEM+ PTX	6.3% (3/48)	‐	3.68	6.72
					FPA008 +Nivolumab +5‐FU	4.8% (2/42)	‐	2.92	5.68
PI3K	Copanlisib/	Nivolumab	NCT03711058	MSS proficient solid tumours	PI3K mutation cohort	9.5% (2/21)	23.8% (5/21)	‐	‐
	BAY80−6946				PI3K wild‐type cohort	0% (0/12)	25.0% (3/12)	‐	‐
	Idelalisib	Pembrolizumab	NCT03257722	NSCLC	‐	0% (0/2)	‐	‐	‐
SIRPα	TTI‐621	Nivolumab	NCT02663518	Hematologic/Solid tumour	SCLC	0% (0/4)	‐	‐	‐
					Hematologic tumour	50% (2/4)	‐	‐	‐
ARG1	INCB001158	Pembrolizumab	NCT02903914	Metastatic solid tumours	INCB001158	1.4% (1/73)	‐	1.9	‐
					INCB001158+Pembrolizumab	11% (13/118)	‐	3	‐
COX2	Aspirin	Ipilimumab	NCT03396952	Cutaneous melanoma	‐	52.2% (12/23)	‐	7.6	8.9
ILT4	MK‐4830	Pembrolizumab	NCT03564691	Advanced solid tumours	MK‐4830+ Pembrolizumab	32.4% (11/34)	‐	‐	‐

Abbreviations: 5‐FU, 5‐fluorouracil; CRC, colorectal cancer; DCR, disease control rate; GEM, gemcitabine; GIST, gastrointestinal stromal tumours; HCC, hepatocellular carcinoma; NSCLC, non‐small‐cell carcinoma; OC, ovarian cancer; ORR, objective response rate; OS, overall survival; PADC, pancreatic ductal adenocarcinomas; PC, pancreatic cancer; PFS, progression‐free survival; pMMR: proficient mismatch repair; PTX, paclitaxel; RCC, renal cell carcinoma; SCLC, small‐cell carcinoma.

### 1.23 IL‐6/STAT3 pathway

IL‐6, a common TAN activator, enhances PD‐L1 expression and suppresses antitumour immunity through the JAK1/STAT3 pathway. Conversely, IL‐6 blockade downregulates PD‐L1 expression.^177^, ^178^, ^179^ Anti‐IL‐6/IL‐6 receptor (IL‐6R) antibodies are being evaluated for their potential to enhance the antitumour activity of ICIs across multiple cancer types.^177^ The anti‐IL‐6R agent tocilizumab has exhibited a favourable safety profile in mitigating cytokine release syndrome linked to ICI treatment.^180^, ^181^ Among ICI‐treated patients with immune‐related adverse events (irAEs), 73% showed resolution of irAEs to grade ≤1 after initiating tocilizumab or IL‐6R antibody sarilumab.^182^ Tocilizumab combined with nivolumab or ipilimumab has demonstrated promising tumour‐suppressive efficacy in melanoma, achieving an ORR of 48.6% (NCT03999749). Additionally, the STAT3 inhibitor napabucasin plus pembrolizumab (NCT02851004) demonstrated encouraging results in metastatic CRC, particularly in patients with high tumour mutational burden. Specifically, in patients with microsatellite instability‐high (MSI‐H) and microsatellite stable (MSS) status, the ORRs were 50% and 10%, respectively.^183^

### 1.24 CSF/CSFR and PI3K pathway

G‐CSF and GM‐CSF in the TME prolong neutrophil survival by upregulating anti‐apoptotic protein Mcl‐1 and inhibiting caspase‐3 through PI3K pathway activation.^184^ GM‐CSF/CSF2R signalling also enhances immunosuppression by upregulating PD‐L1 and FATP2 via STAT5 pathway.^185^ A phase I/II trial in melanoma showed that sargramostim (a GM‐CSF inhibitor) combined with ipilimumab extended median survival by 5 months compared with ipilimumab monotherapy.^186^ Multiple CSF‐1R inhibitors (axatilimab, pexidartinib/PLX3397, ARRY‐382, BLZ945, AMG820, cabiralizumab) combined with ICIs have entered clinical trials for solid tumours. Pexidartinib plus pembrolizumab achieved an ORR of 15.4% in melanoma (NCT02452424), while cabiralizumab plus nivolumab yielded ORRs of 13.8% and 13.3% in HNSCC and OC, respectively (NCT02526017). However, the combination of CSF‐1R inhibitors and ICIs exhibited limited efficacy in NSCLC, PDAC, RCC and CRC, with ORRs below 10%. Although AMG 820 plus pembrolizumab was well‐tolerated in a trial for specific advanced solid tumours (NCT02713529), only 2.6% of patients exhibited immune‐related partial responses.^187^ Further results from ongoing trials are awaited to clarify the efficacy of CSF‐1R inhibitor combinations. Additionally, twelve clinical trials exploring PI3K inhibitors (copanlisib, idelalisib, itacitinib, and axatilimab) combined with ICIs to inhibit TAN activation. Copanlisib in combination with nivolumab demonstrated antitumour efficacy in patients with PI3K‐mutant tumours but poor efficacy in PI3K‐wild‐type tumours (NCT03711058). Antitumour activity has also been observed for eganelisib in patients who progressed on prior ICI treatment (NCT02637531).^188^

### 1.25 CD47‐SIRPα axis

Researches are also focused on enhancing neutrophil antitumour responses. The CD47‐signal regulatory protein α (SIRPα) axis represents a promising target for combination targeted therapy.^189^ Cancer cells evade neutrophil‐mediated immune clearance through CD47‐SIRPα interactions. Blocking this signalling induces an anti‐tumour phenotype of TANs, promoting antibody‐dependent cellular cytotoxicity (ADCC) and activating T cell responses.^190^, ^191^ Clinical trials of anti‐CD47 (ALX148/evorpacept, AO‐176, magrolimab) or anti‐SIRPα (TTI‐621, TJ011133, BI765063) antibodies combined with ICIs are currently assessing the efficacy of this combination. A phase I trial of ALX148 plus pembrolizumab (NCT03013218) reported acceptable safety in advanced solid tumours,^192^ and TTI‐621 combined with nivolumab achieved a 50% ORR in 4 Hodgkin lymphoma patients (NCT02663518), demonstrating the promise of targeting CD47‐SIRPα signalling.^193^

### 1.26 Soluble inhibitory factor

Among strategies targeting immunosuppressive factors, the Arg inhibitor INCB001158 was well‐tolerated as a single agent or combined with pembrolizumab for advanced solid malignancies (NCT02903914), though the response rates did not exceed historical controls.^194^ Targeting COX‐2 to block PGE2 release or inhibiting EP2/EP4 to block PGE2 is another effective approach. In preclinical tumour models, COX inhibitors combined with pembrolizumab improved ICB efficacy and reversed resistance.^195^ The result of a retrospective study also supports the positive impact of concurrent use of low‐dose aspirin (a COX inhibitor) on outcomes during ICI treatment.^196^ Trials of COX inhibitors (aspirin, celecoxib) and EP2/EP4 receptor antagonists (grapiprant, OKN4395, TPST‐1495) plus ICIs for solid tumours are ongoing. Targeting S100A9, tasquinimod can sensitize BRCA1‐mutated breast cancer to ICB.^197^ Although several non‐specific S100A8/A9 inhibitors are under clinical evaluation, no specific S100A8/A9 inhibitor combined with ICIs has entered clinical trials.

For oxidative inhibitory factors, inhibition of immunoglobulin‐like transcript 4 (ILT4) reduces ROS release and T cell suppression by impairing neutrophil phagocytosis and respiratory burst.^198^ The ILT4 inhibitor MK‐4830 plus pembrolizumab achieved a 32.4% ORR in advanced solid tumours (NCT03564691), with nearly 50% of responders having prior ICI resistance. Combination therapy demonstrated higher T cell activation than ICI monotherapy, positioning ILT4 inhibitors as a promising new immunotherapy.^198^ The nitric oxide synthase inhibitor L‐NMMA reduces NO production, mitigating TAN‐induced T cell apoptosis and chemotaxis defects. Biopsies from triple‐negative breast cancer patients showed reduced N2‐TAN markers after L‐NMMA treatment,^199^ and L‐NMMA combined with pembrolizumab is being examined in trials for precision therapy in NO‐hypersecreting tumours (NCT03236935).

### 1.27 Targeting angiogenesis

Across clinical investigations evaluating neutrophil‐directed anti‐angiogenic agents in combination with ICIs, 81 studies focus on macromolecular single‐target angiogenesis inhibitors. Bevacizumab (anti‐VEGF‐A monoclonal antibody) dominates this landscape, accounting for 80 studies (98.8%), with only one study involving aflibercept. A total of 230 trials investigate combinations of tyrosine kinase inhibitors (TKIs) with ICIs, including 149 trials evaluating lenvatinib. Lenvatinib blocks both classical and compensatory angiogenic pathways targeting VEGFR2/3, FGFR and other pro‐angiogenic receptors.^200^ Beyond lenvatinib, 34 trials include regorafenib, 66 involve cabozantinib, and other agents like axitinib (23 trials), anlotinib (16 trials), sunitinib (13 trials), and sorafenib are also under evaluation. Additionally, a phase I trial (NCT03239145) is evaluating the ANGPT1/2 inhibitor trebananib plus pembrolizumab for advanced solid tumours. Although neutrophils contribute only partially to tumour angiogenesis, they exert a unique role in modulating the pro‐angiogenic microenvironment through the secretion of factors like VEGF and MMP‐9, and via NET formation. Currently, the vast majority of anti‐angiogenic regimens rely on pan‐angiogenesis inhibition. Precision strategies targeting TAN‐specific angiogenesis still await further development.

### 1.28 Targeting NETs

NETs act as critical immunosuppressive mediators in the TME by forming physical barriers, releasing immunosuppressive factors, and promoting angiogenesis. Strategies targeting NET formation and degradation offer new directions for relieving T cell inhibition (Table S5).

### 1.29 NETs formation

NET formation relies on NADPH oxidase‐mediated ROS burst and peptidylarginine deiminase 4 (PAD4)‐induced histone citrullination.^201^, ^202^, ^203^ The ILT4 inhibitor MK‐4830, by reducing ROS production, can suppress NET formation, thereby alleviating ICI resistance. The PAD4 inhibitor JBI‐589 blocks neutrophil chemotaxis by downregulating CXCR2 expression, significantly increasing activated CD8^+^T cell infiltration and enhancing ICB efficacy in mouse models.^204^ Other PAD4 inhibitors, such as Cl‐amidine and GSK48, also exhibit promising antitumour activity in spontaneous melanoma mouse models.^205^ However, the short serum half‐life of currently available PAD4 inhibitors severely limits their clinical application.

Cytokines or chemokines can induce NET extrusion via CXCR1/2 signalling, making CXCR1/2 inhibitors a dual strategy to suppress neutrophil chemotaxis and reduce NET production.^67^ Metformin, recently identified as an immunomodulator of neutrophils, decreases NET formation by blocking PKCIIβ membrane translocation and NADPH oxidase activity independently of its glucose‐lowering effects.^206^, ^207^, ^208^ In non‐alcoholic steatohepatitis‐related liver tumours, metformin rescues impaired CD8^+^T cell activity, thereby reinstating the therapeutic response to ICIs.^209^ A retrospective meta‐analysis links metformin‐ICI combination therapy to improved outcomes in lung cancer patients.^210^ In an HNSCC trial (NCT03618654), metformin plus durvalumab reduced Foxp3^+^Treg and elevated CD8^+^T cell infiltration in tumour foci compared with durvalumab monotherapy.^211^ In metastatic microsatellite stable CRC (NCT03800602), metformin plus nivolumab achieved a DCR of only 11.1%. Another trial in patients with unresectable NSCLC (NCT03048500) reported an ORR of merely 5.9% for this combination. Although metformin combined with immune checkpoint inhibitors demonstrates no significant efficacy, this treatment trended to increasing tumour T cell infiltration, indicating its potential for TME remodelling.^212^

### 1.30 NETs digestion

Deoxyribonuclease (DNase) disrupts the physical barrier and signalling molecule carrier functions of NETs by hydrolyzing NETs‐DNA.^213^ DNase I‐mediated NETs degradation reduces intratumoral TAN infiltration and increases cytotoxic T cell infiltration.^95^, ^214^ Combination with anti‐PD‐L1 therapy restores T cell function and significantly reduces tumour metastasis.^70^ However, the short circulating half‐life of DNase I requires multiple intravenous injections.^215^, ^216^, ^217^ Only a few clinical trials have evaluated recombinant DNase (pulmozyme, Oshadi D) in tumour patients (NCT00536952, NCT02462265), showing antitumour activity and safety, though its combination with ICIs awaits efficacy validation.

### 1.31 Challenges in targeting neutrophils

While targeting TANs provides a novel rationale for overcoming ICI resistance, its clinical application is confronted with dual core challenges, including systemic risks induced by the intervention and the high intrinsic heterogeneity of the target cells. This necessitates an evolution of therapeutic strategy from ‘broad intervention’ towards ‘precise modulation’.

The primary clinical challenge focuses on treatment safety. As neutrophils are cornerstone effectors of innate immune defence, systemically inhibiting their function or depleting their numbers inevitably compromises the host's anti‐infective capacity. Strategies directly targeting neutrophil production and survival readily induce neutropenia, significantly increasing the risk of opportunistic infections.^135^, ^218^ Drugs targeting molecules broadly expressed on cell surfaces, such as anti‐CD47 antibodies, can cause haematological toxicities like anaemia due to ‘off‐target’ effects.^192^, ^193^, ^219^ Even interventions that do not directly clear cells, such as modulating their recruitment (e.g., CXCR1/2 inhibitors) or functional activation (e.g., PI3K inhibitors), may disrupt immune homeostasis, leading to complex side effects like liver function abnormalities and fatigue.^187^, ^188^ These toxicities might interact with the immune‐related adverse events inherent to ICIs, further complicating clinical management.

A more profound challenge stems from the intrinsic functional plasticity and phenotypic heterogeneity of TANs. Neutrophils within the TME are not a homogeneous population but exist as a continuum of phenotypes, dynamically differentiating under various signalling cues into subsets with pro‐tumorigenic or potential anti‐tumour functions. This heterogeneity creates therapeutic dilemmas. Indiscriminate cell depletion risks eliminating not only immunosuppressive subsets but also those with antigen‐presenting or direct cytotoxic potential, potentially yielding unintended pro‐metastatic consequences.^220^ Besides, many current targets, such as S100A8/A9 and COX2, are markers of activation rather than specific subtype identifiers, leading to insufficient intervention precision and a lack of subtype selectivity for drugs. Furthermore, blocking a specific pathway, such as TGF‐β, may trigger the compensatory recruitment of other immunosuppressive cells or drive neutrophils towards alternative inhibitory phenotypes, resulting in treatment resistance.^221^

### 1.32 Future perspectives

Given the clinical challenges in neutrophil‐targeted therapy, enhancing intervention precision has become an urgent imperative. The exploration of predictive biomarkers for neutrophil‐dependent resistance is crucial. Baseline metrics such as the NLR or the proportion of PD‐L1^+^ TANs could serve as initial screening tools to identify TAN‐dominated immunosuppression and preliminarily predict treatment responses. Advanced profiling via single‐cell sequencing to decipher TAN phenotypes, NETs burden, and key pathway activity within the TME could enable precise patient stratification, particularly for those with high TAN infiltration. Furthermore, following drug administration, dynamic monitoring of NLR, circulating immunosuppressive factor levels and the proportion of PD‐L1^+^ TANs can help uncover the emergence of adaptive immunosuppressive responses and acquired resistance.^222^ Integrating these dynamic indicators facilitates the construction of models for efficacy prediction and early resistance detection, thereby providing real‐time guidance for personalized treatment adaptation.

Developing novel, precise therapeutics focused on TAN‐specific molecules may effectively avoid the infection risks associated with pan‐neutrophil suppression. Leveraging the plasticity of TAN function, creating biomarker‐guided drug delivery systems or bispecific antibodies could enable selective modulation of pro‐tumorigenic TAN subsets. Optimizing the sequence and harnessing mechanistic synergy in combination therapies are also exploration directions for improving efficacy. Currently, most early‐phase clinical trials adopt a concurrent dosing strategy to evaluate drug synergies. For instance, IL‐6 inhibitors, while suppressing neutrophil activation, can also alleviate adverse reactions induced by ICIs.^179^, ^180^ This immediate mechanistic synergy makes concurrent administration an advantageous choice. In contrast, a sequential dosing strategy aims to first remodel the immunosuppressive TME to create favourable conditions for subsequent ICI efficacy. In a phase I/II clinical trial for nasopharyngeal carcinoma, sequential therapy with a TGF‐β inhibitor followed by a PD‐1 inhibitor achieved an ORR of 26.1%.^223^ This ‘reprogram‐then‐block’ approach may enhance efficacy by using the TGF‐β inhibitor to reverse the pro‐tumour phenotype of TANs before initiating ICI therapy. Sequential therapy may represent a promising dosing strategy for tumours with high TAN burden, a dominant pro‐tumour TAN phenotype, or in scenarios where the combined toxicity profile is a significant concern. Future optimization of treatment timing will likely require an integrated consideration of the specific drug's mechanism of action and the baseline characteristics of the TME to achieve truly personalized therapy. Addressing challenges like the short half‐life and low tissue penetration of some NET‐targeting drugs, future research should focus on developing reversible PAD4 inhibitors and employing nanocarriers to improve tumour tissue delivery efficiency and reduce systemic toxicity.

Future research must remain closely aligned with the mechanistic understanding of TAN heterogeneity. Through multi‐dimensional technological breakthroughs and optimization, the field can advance TAN‐targeted combination therapies from ‘classical combination’ to ‘precise synergy,’ offering a new paradigm for overcoming ICI resistance and expanding the population eligible for cancer immunotherapy.

## 2 CONCLUSION

The mechanisms of ICI resistance mediated by neutrophils highlight the complexity of tumour microenvironment regulation. The diversification of targeted strategies provides multiple dimensions for overcoming drug resistance. Future research should closely integrate fundamental mechanisms analysis with clinical translation needs. Through biomarker‐driven precision medicine and optimization of mechanisms with multi‐target synergy, TANs can be transformed from ‘drivers of drug resistance’ into ‘activators of immune responses’. Despite the challenges in target specificity, toxicity management, and individualized treatment, with a deeper understanding of TAN heterogeneity and dynamic regulation, neutrophil‐centred combination therapies are expected to become a cornerstone of tumour immunotherapy, bringing survival benefits to more patients.

## AUTHOR CONTRIBUTIONS

**Ying Ning**: Conceptualization; investigation; writing—original draft; funding acquisition. **Ke Lei**: Writing—review & editing. **Xinyan Gao, Yan Kong and Yuping Shan**: Investigation; validation. **Tian Tian**: Writing—review & editing. **Zhumei Cui**: Writing—review & editing; funding acquisition. **He Ren**: Supervision; writing—review & editing; project administration.

## CONFLICT OF INTEREST STATEMENT

The authors declare no conflict of interest.

## ETHICS STATEMENT

Ethical approval is not applicable to this review article.

## Supporting information



Supporting Information

Supporting Information

## Data Availability

The data supporting the findings of this study are available from the corresponding author upon reasonable request.
