# Supplementary material for: Targeting neutrophil‐driven immunosuppression: A strategy to overcome immune checkpoint inhibitor resistance
Source: Clin Transl Med. 2026 Jan 5;16(1):e70582. doi: 10.1002/ctm2.70582 (PMC12771606; doi:10.1002/ctm2.70582)
Supplement: Supplementary file 1 — Supporting Information [file CTM2-16-e70582-s001.docx]

| **Table S1 Studies on NLR in tumor patients treated with ICIs** | | | | | |
| --- | --- | --- | --- | --- | --- |
| **Drug** | **Caner type** | **Number of cases** | **Prognostic indicators related to NLR** | **Threshold** | **DOI of reference** |
| **Durvalumab** | **NSCLC** | **154** | **PFS, OS** | **dNLR>3** | **10.1038/s41598-024-70214-y** |
| **Durvalumab** | **Stage III NSCLC** | **84** | **PFS** | **NLR≥3 or NLR≥5** | **10.1111/1759-7714.14667** |
| **Durvalumab** | **Stage III unresectable NSCLC** | **145** | **OS, PFS** | **-** | **10.1111/1759-7714.14646** |
| **Durvalumab** | **NSCLC** | **113** | **PFS** | **-** | **10.1016/j.radonc.2021.12.016** |
| **Durvalumab** | **Stage III unresectable NSCLC** | **31** | **PFS** | **-** | **10.1111/1759-7714.13426** |
| **Durvalumab** | **Stage III NSCLC** | **821** | **OS** | **-** | **10.1016/j.lungcan.2021.11.021** |
| **Durvalumab** | **Unresectable Stage III NSCLC** | **472** | **OS** | **-** | **10.1007/s11523-024-01111-7** |
| **Durvalumab** | **Locally Advanced NSCLC** | **105** | **PFS** | **NLR≥4.3** | **10.1016/j.prro.2020.09.010** |
| **Ipilimumab** | **Melanoma** | **38** | **PFS** | **NLR>3.4** | **10.1248/BPB.B22-00750** |
| **Ipilimumab** | **Metastatic Melanoma** | **183** | **OS** | **NLR≥4** | **10.1002/cam4.878** |
| **Ipilimumab** | **Metastatic Melanoma** | **187** | **OS, PFS** | **NLR≥5** | **10.1038/bjc.2015.180** |
| **Ipilimumab** | **Unresectable Stage III/IV Melanoma** | **197** | **OS, PFS** | **-** | **10.1016/j.ebiom.2017.03.029** |
| **Ipilimumab** | **Melanoma** | **58** | **OS** | **NLR≥4** | **10.1111/BJD.14155** |
| **Ipilimumab** | **Melanoma** | **104** | **OS** | **NLR≥5** | **10.4143/crt.2016.024** |
| **Nivolumab** | **NSCLC** | **62** | **RR** | **dNLR** | **10.1002/JCP.26609** |
| **Nivolumab** | **Advanced HCC** | **45** | **RR** | **NLR＞2.5** | **10.3390/cancers13071607** |
| **Nivolumab** | **GC** | **98** | **OS** | **NLR＞3** | **10.1007/s00280-019-04023-w** |
| **Nivolumab** | **Advanced NSCLC** | **44** | **OS, PFS** | **NLR＞3.07** | **10.1002/jcla.22964** |
| **Nivolumab** | **Recurrent/Metastatic HNSCC** | **124** | **OS** | **NLR>3.5** | **10.1007/s00405-024-08744-4** |
| **Nivolumab** | **Unresectable Advanced/Recurrent GC** | **111** | **OS, PFS** | **NLR≥1.4** | **10.1186/s12885-024-12813-6** |
| **Nivolumab** | **Advanced RCC** | **571** | **OS, RR** | **NLR≥3.2** | **10.1007/s00262-024-03741-2** |
| **Nivolumab** | **Deficient Mismatch Repair GC** | **96** | **PFS** | **NLR≥3.8** | **10.1007/s10120-024-01509-2** |
| **Nivolumab** | **Advanced HCC** | **189** | **OS, HPD** | **NLR≥4.125** | **10.1016/j.jhep.2020.08.010** |
| **Nivolumab** | **Advanced Melanoma** | **97** | **OS, PFS** | **NLR≥4.7** | **10.1186/s40425-018-0383-1** |
| **Nivolumab** | **Advanced GC** | **26** | **OS** | **NLR≥5** | **10.18632/oncotarget.26145** |
| **Nivolumab** | **Recurrent OSCC** | **64** | **OS** | **NLR≥5** | **10.1016/j.bjoms.2023.03.012** |
| **Nivolumab** | **Recurrent or Metastatic HNSCC** | **29** | **OS** | **NLR≥5** | **10.1080/00016489.2019.1699250** |
| **Nivolumab** | **Advanced HCC** | **103** | **OS** | **NLR≥5** | **10.1002/cam4.3135** |
| **Nivolumab** | **NSCLC** | **187** | **OS, PFS** | **NLR≥5** | **10.1007/s12325-020-01229-w** |
| **Nivolumab** | **Metastatic RCC** | **65** | **OS, PFS** | **NLR≥5** | **10.1007/s10147-019-01528-5** |
| **Nivolumab** | **NSCLC** | **175** | **OS, PFS** | **NLR≥5** | **10.1016/j.lungcan.2017.01.013** |
| **Nivolumab** | **Advanced NSCLC** | **52** | **OS** | **NLR≥5.39** | **10.1016/j.cllc.2018.04.021** |
| **Nivolumab** | **Metastatic RCC** | **38** | **OS, PFS** | **NLR≥5.5** | **10.1016/j.clgc.2017.12.015** |
| **Nivolumab** | **Recurrent/Metastatic HNSCC** | **39** | **OS** | **NLR≥7** | **10.3390/cancers12082299** |
| **Nivolumab** | **Advanced GC** | **29** | **RR** | **NLR at 4w** | **10.1007/S00595-020-02048-W** |
| **Nivolumab** | **Metastatic RCC and NSCLC** | **161** | **OS, PFS** | **NLR at 6w** | **10.1007/s00262-020-02637-1** |
| **Nivolumab** | **NSCLC** | **109** | **OS** | **NLR≥5 at 2w** | **10.1371/journal.pone.0197743** |
| **Nivolumab** | **Metastatic Uveal Melanoma** | **14** | **OS, PFS** | **NLR≥5 at 6w** | **10.1097/CMR.00000000000000617** |
| **Nivolumab** | **Metastatic RCC** | **422** | **OS, PFS, HPD** | **ΔNLR** | **10.3389/fonc.2022.955501** |
| **Nivolumab** | **Advanced NSCLC** | **59** | **OS** | **ΔNLR>1** | **10.1371/journal.pone.0219060** |
| **Nivolumab** | **NSCLC** | **52** | **OS** | **-** | **10.1016/j.lungcan.2017.07.024** |
| **Nivolumab** | **Metastatic HNSCC** | **56** | **OS** | **-** | **10.18926/AMO/62228** |
| **Nivolumab** | **Recurrent/Metastatic HNSCC** | **164** | **OS** | **-** | **10.21873/invivo.12780** |
| **Nivolumab** | **Advanced GC** | **72** | **OS** | **-** | **10.1007/s10147-020-01810-x** |
| **Nivolumab** | **SGC** | **24** | **OS** | **-** | **10.1038/s41598-020-73965-6** |
| **Nivolumab** | **NSCLC** | **108** | **OS** | **-** | **10.3389/fonc.2021.625668** |
| **Nivolumab** | **Advanced HCC** | **81** | **OS, PFS** | **-** | **10.1177/17588359221113266** |
| **Nivolumab** | **Advanced ESCC** |  | **OS, PFS** | **-** | **10.2147/OTT.S404926** |
| **Nivolumab** | **NSCLC** | **30** | **PFS** | **-** | **10.1111/1759-7714.12838** |
| **Nivolumab** | **Unresectable or Recurrent GC** | **44** | **OS** | **-** | **10.3892/ol.2024.14766** |
| **Nivolumab** | **NSCLC** | **19** | **OS** | **-** | **10.1371/journal.pone.0193018** |
| **Nivolumab** | **NSCLC** | **101** | **OS** | **NLR≥3** | **10.1007/s10147-018-1250-2** |
| **Nivolumab** | **Metastatic NSCLC** | **65** | **OS** | **-** | **10.2217/imt-2019-0154** |
| **Nivolumab** | **Advanced Melanoma** |  | **OS** | **-** |  |
| **Nivolumab** | **GC** |  | **OS** | **NLR>2.5** | **10.1007/s11523-020-00716-y** |
| **Nivolumab** | **Metastatic RCC** | **58** | **OS** | **NLR≥3** | **10.1007/s11523-019-00660-6** |
| **Nivolumab** | **NSCLC** | **201** | **PFS** | **NLR≥4** | **10.1002/cam4.1234** |
| **Nivolumab** | **NSCLC** | **15** | **RR** | **NLR≥5 at 4-6w** | **10.1111/1759-7714.12952** |
| **Nivolumab** | **Advanced NSCLC** | **213** | **OS** | **ΔNLR≥1** | **10.1111/1759-7714.14303** |
| **Nivolumab** | **Metastatic Clear Cell RCC** | **90** | **HPD** | **-** | **10.1186/s40425-018-0425-8** |
| **Nivolumab** | **Recurrent/Metastatic HNSCC** | **102** | **OS** | **-** | **10.1002/cam4.3124** |
| **Nivolumab** | **NSCLC** | **120** | **OS** | **-** | **10.21873/anticanres.13048** |
| **Nivolumab** | **Metastatic RCC** | **39** | **PFS** | **-** | **10.1007/s10147-020-01708-8** |
| **Nivolumab** | **Recurrent or Metastatic HNSCC** | **41** | **RR** | **-** | **10.1002/hed.25737** |
| **Nivolumab** | **Advanced Melanoma** | **124** | **OS** | **-** | **10.1093/jjco/hyab064** |
| **Pembrolizumab** | **Advanced/Recurrent EC** | **25** | **OS** | **NLR≥5.39** | **10.1111/jog.16182** |
| **Pembrolizumab** | **NSCLC** | **221** | **OS, PFS, ORR** | **dNLR≥2.6** | **10.1136/jitc-2021-003536** |
| **Pembrolizumab** | **Advanced NSCLC** | **83** | **OS** | **NLR>2** | **10.21037/TLCR-21-100** |
| **Pembrolizumab** | **Advanced UC** | **41** | **OS** | **NLR>3** | **10.3390/cancers15245780** |
| **Pembrolizumab** | **Advanced NSCLC** | **102** | **OS** | **NLR>3.1** | **10.3389/fonc.2021.752545** |
| **Pembrolizumab** | **Metastatic UC** | **543** | **OS** | **NLR≥3** | **10.1111/iju.14861** |
| **Pembrolizumab** | **Stage IV NSCLC** | **339** | **OS** | **NLR≥3** | **10.3389/fonc.2024.1341084** |
| **Pembrolizumab** | **Metastatic UC** | **393** | **OS** | **NLR≥3** | **10.1111/iju.70002** |
| **Pembrolizumab** | **Advanced NSCLC** | **845** | **OS** | **NLR≥4** | **10.1007/s00262-022-03232-2** |
| **Pembrolizumab** | **Advanced UC** | **41** | **OS** | **NLR at 6w** | **10.1007/s10147-019-01613-9** |
| **Pembrolizumab** | **NSCLC** | **119** | **OS, PFS** | **NLR>5** | **10.5582/bst.2019.01279** |
| **Pembrolizumab** | **Recurrent and/or Metastatic HNSCC** | **87** | **RR** | **NLR>6** | **10.1007/s10637-023-01388-x** |
| **Pembrolizumab** | **NSCLC** | **132** | **OS, PFS** | **NLR≥5** | **10.21037/tlcr-19-583** |
| **Pembrolizumab** | **Recurrent/Metastatic HNSCC** | **74** | **OS, PFS** | **NLR≥5** | **10.1038/s41598-024-79130-7** |
| **Pembrolizumab** | **NSCLC** | **236** | **OS, PFS** | **NLR≥5** | **10.1111/1759-7714.15036** |
| **Pembrolizumab** | **Recurrent EC** | **106** | **OS** | **NLR≥6** | **10.1016/j.ygyno.2022.07.010** |
| **Pembrolizumab** | **Advanced NSCLC** | **220** | **OS** | **-** | **10.21037/tlcr-20-541** |
| **Pembrolizumab** | **NSCLC** | **51** | **OS** | **-** | **10.21873/anticanres.13902** |
| **Pembrolizumab** | **Metastatic NSCLC** | **136** | **OS** | **-** | **10.1016/j.cllc.2022.03.010** |
| **Pembrolizumab** | **Elderly Patients with Non-squamous NSCLC** | **30** | **OS** | **-** | **10.2169/internalmedicine.3649-24** |
| **Pembrolizumab** | **Metastatic UC** | **198** | **OS** | **-** | **10.1007/s10147-021-02046-z** |
| **Pembrolizumab** | **Metastatic NSCLC** | **128** | **OS, PFS** | **-** | **10.1111/1759-7714.14256** |
| **Pembrolizumab** | **Recurrent/Metastatic SGC** | **20** | **OS, PFS** | **-** | **10.1002/hed.27565** |
| **Pembrolizumab** | **Stage IV/Recurrent NSCLC** | **51** | **OS, PFS** | **-** | **10.21037/TLCR-21-156** |
| **Pembrolizumab** | **Recurrent/Metastatic HNSCC** | **147** | **OS, ORR, DCR** | **-** | **10.21873/anticanres.17235** |
| **Pembrolizumab** | **Advanced NSCLC** | **47** | **PFS** | **-** | **10.3390/PH15111407** |
| **Pembrolizumab** | **Advanced NSCLC** | **52** | **PFS** | **-** | **10.1177/17588359211068732** |
| **Pembrolizumab** | **Advanced UC** | **101** | **PFS** | **-** | **10.1111/iju.15133** |
| **Pembrolizumab** | **Advanced ESCC** | **55** | **OS** | **-** | **10.3748/wjg.v29.i41.5641** |
| **Pembrolizumab** | **NSCLC with Bone Metastasis** | **110** | **OS** | **NLR>5.55** | **10.21037/TLCR-21-1033** |
| **Pembrolizumab** | **UC** | **458** | **OS, PFS** | **NLR≥3** | **10.1007/s00262-021-03000-8** |
| **Pembrolizumab** | **Metastatic UC** | **160** | **OS** | **NLR≥3.24** | **10.3390/biomedicines10071609** |
| **Pembrolizumab** | **Recurrent/Metastatic HNSCC** | **29** | **OS, PFS** | **NLR≥4.5** | **10.1002/hed.27671** |
| **Pembrolizumab** | **OSCC** | **52** | **OS, PFS** | **-** | **10.1002/ohn.1088** |
| **Pembrolizumab** | **UC** | **50** | **OS, PFS** | **-** | **10.1002/cam4.4779** |
| **Pembrolizumab** | **Recurrent/Metastatic HNSCC** | **54** | **OS, PFS** | **-** | **10.3390/cancers16234056** |
| **Pembrolizumab** | **Colorectal Cancer** | **62** | **RR** | **-** | **10.1038/s41467-022-35096-6** |
| **Pembrolizumab** | **HCC** | **60** | **RR** | **-** | **10.1186/s13073-021-00995-8** |
| **Pembrolizumab**  **/Atezolizumab** | **Advanced NSCLC** | **241** | **OS** | **-** | **10.7150/jca.80517** |
| **Pembrolizumab** | **Thyroid Cancers** | **57** | **PFS** | **-** | **10.1158/1078-0432.CCR-23-3417** |
| **Pembrolizumab** | **Recurrent/Metastatic HNSCC and SGC** | **50** | **OS, PFS** | **-** | **10.1002/hed.27252** |
| **Durvalumab/Atezolizumab** | **NSCLC** | **72** | **OS** | **NLR>4** | **10.3390/vaccines12050474** |
| **Ipilimumab+Nivolumab** | **Advanced RCC** | **84** | **OS** | **NLR≥4.8 at ninth week** | **10.1007/s10147-024-02593-1** |
| **Ipilimumab+Nivolumab** | **Metastatic Melanoma** | **44** | **OS** | **NLR≥5** | **10.1007/s12094-021-02692-9** |
| **Ipilimumab+Nivolumab** | **Advanced RCC** | **116** | **OS** | **ΔNLR≥25%** | **10.1007/s10238-024-01544-4** |
| **Ipilimumab+Nivolumab** | **Esophageal Cancer** | **27** | **OS** | **-** | **10.3389/fonc.2024.1449941** |
| **Ipilimumab+Nivolumab** | **Melanoma** | **68** | **PFS** | **-** | **10.1111/1346-8138.15073** |
| **Ipilimumab+Nivolumab** | **Metastatic RCC** | **43** | **OS** | **NLR＞2.8** | **10.3390/jcm10225325** |
| **Ipilimumab+Nivolumab** | **Advanced or Metastatic RCC** | **35** | **OS** | **NLR＞4.6** | **10.3390/curroncol28020133** |
| **Ipilimumab+Nivolumab** | **Metastatic RCC** | **110** | **OS** | **NLR≥3.2** | **10.1186/s40364-021-00334-4** |
| **Ipilimumab+Nivolumab** | **Melanoma with Brain Metastasis** | **84** | **OS** | **NLR≥4** | **10.1097/CJI.0000000000000385** |
| **Ipilimumab+Nivolumab** | **Advanced NSCLC** | **101** | **OS** | **-** | **10.1159/000534169** |
| **Ipilimumab+Nivolumab** | **Advanced RCC/Melanoma** | **71** | **OS** | **NLR≥3** | **10.3390/biomedicines10112758** |
| **Ipilimumab+Nivolumab** | **Advanced Melanoma** | **209** | **OS** | **-** | **10.1002/cam4.1356** |
| **Nivolumab/Atezolizumab** | **KRAS-Mutated Patients with NSCLC** | **26** | **OS** | **NLR>3.5** | **10.3390/curroncol30010037** |
| **Nivolumab/Pembrolizumab** | **Advanced NSCLC** | **83** | **OS, Recurrence** | **NLR≥5** | **10.3390/curroncol30120769** |
| **Nivolumab/Pembrolizumab** | **Advanced ESCC** |  | **OS, PFS** | **ΔNLR>1.4fold** | **10.4143/crt.2020.1198** |
| **Nivolumab/Pembrolizumab** | **Gastrointestinal Cancer** | **61** | **OS** | **-** | **10.21873/anticanres.15781** |
| **Nivolumab/Pembrolizumab** | **NSCLC** | **44** | **OS** | **-** | **10.21037/jtd.2019.04.41** |
| **Nivolumab/Pembrolizumab** | **Recurrent/Metastatic OSCC** | **31** | **OS** | **-** | **10.1016/j.jormas.2024.101961** |
| **Nivolumab/Pembrolizumab** | **Locally Advanced/Metastatic GC** | **54** | **OS** | **-** | **10.3389/fonc.2022.976078** |
| **Nivolumab/Pembrolizumab** | **Recurrent/Metastatic HNSCC** | **36** | **OS, PFS** | **-** | **10.1007/s12672-023-00774-4** |
| **Nivolumab/Pembrolizumab/Atezolizumab** | **Advanced/Metastatic NSCLC** | **166** | **OS** | **NLR≥4** | **10.1007/s00432-022-04215-7** |
| **Nivolumab/Pembrolizumab**  **/Atezolizumab** | **NSCLC** | **73** | **OS, Recurrence** | **NLR≥5.6** | **10.21873/anticanres.15158** |
| **Nivolumab/Pembrolizumab** | **Advanced NSCLC** | **259** | **OS** | **-** | **10.3389/fonc.2021.770268** |
| **ICI** | **Advanced NSCLC** | **108** | **RR** | **NLR≥3 at 6 week** | **10.3390/cancers15102733** |
| **ICI** | **NSCLC** | **33** | **PFS, OS** | **NLR≥4.9** | **10.1007/s00432-020-03150-9** |
| **ICI** | **NSCLC with Bone Metastasis** | **142** | **OS** | **NLR>5** | **10.3389/fimmu.2021.697298** |
| **ICI** | **Advanced NSCLC** | **89** | **PFS** | **NLR≥1** | **10.21037/jtd-20-3416** |
| **ICI** | **NSCLC** | **1234** | **PFS** | **NLR≥4** | **10.3390/cancers16101811** |
| **ICI** | **Advanced EC** | **35** | **PFS** | **NLR≥4.92** | **10.1093/jjco/hyae123** |
| **ICI** | **Small Cell Lung Cancer** | **111** | **OS** | **-** | **10.1177/17588359221097191** |
| **ICI** | **Advanced RCC** | **35** | **PFS** | **-** | **10.3332/ecancer.2024.1753** |
| **NSCLC: Non-Small Cell Lung Cancer; HCC: Hepatocellular Carcinoma; HNSCC: Head and Neck Squamous Cell Carcinoma; GC: Gastric Carcinoma; RCC: Renal Cell Carcinoma; OSCC: Oral Squamous Cell Carcinoma; UC: Urothelial Carcinoma; ESCC: Esophageal Squamous Cell Carcinoma; SGC: Salivary Gland Cancer; EC: Endometrial Cacinoma; OS: Over-all Survival; PFS: Progression-Free Survival; ORR: Objective Response Rate; RR: Response Rate; HPD: Hyperprogressive Disease** | | | | | |
