# Supplementary material for: Targeting neutrophil‐driven immunosuppression: A strategy to overcome immune checkpoint inhibitor resistance
Source: Clin Transl Med. 2026 Jan 5;16(1):e70582. doi: 10.1002/ctm2.70582 (PMC12771606; doi:10.1002/ctm2.70582)
Supplement: Supplementary file 2 — Supporting Information [file CTM2-16-e70582-s002.docx]

| **Table S2. Clinical Trials of ICIs Combined with Drugs Targeting Neutrophil Recruitment** | | | | | | |
| --- | --- | --- | --- | --- | --- | --- |
| **Target** | **Drug** | **ICIs** | **Trial Number** | **Cancer Type** | **Phase** | **State** |
| **CXCR1/2** | **SX-682** | **Pembrolizumab** | **NCT03161431** | **Advanced Melanoma** | **I** | **Recruiting** |
|  |  |  | **NCT05570825** | **Metastasis / Recurrent NSCLC** | **II** | **Recruiting** |
|  |  | **Nivolumab** | **NCT04599140** | **Refractory RAS Mutated MSS CRC** | **I/II** | **Recruiting** |
|  |  |  | **NCT04477343** | **PDAC** | **I** | **Recruiting** |
| **IL-8/CXCL8** | **BMS-986253** | **Nivolumab** | **NCT04123379** | **NSCLC/HNSCC** | **II** | **Active** |
|  |  |  | **NCT03689699** | **PC** | **I/II** | **Active** |
|  |  |  | **NCT04050462** | **HCC** | **II** | **Active** |
|  |  |  | **NCT04572451** | **Advanced Solid Tumors/Melanoma** | **II** | **Recruiting** |
|  |  | **Nivolumab/Ipilimumab** | **NCT03400332** | **Cancer/Melanoma** | **I/II** | **Active** |
| **CXCL12** | **NOX-A12/**  **Olaptesed** | **Pembrolizumab** | **NCT03168139** | **Metastasis CRC** | **Ib/II** | **Completed** |
|  |  |  | **NCT04901741** | **PDAC** | **II** | **Completed** |
| **CCR2/5** | **BMS-813160** | **Nivolumab** | **NCT03184870** | **Metastasis CRC/PDAC** | **I/II** | **Completed** |
|  |  |  | **NCT04123379** | **NSCLC/HNSCC** | **II** | **Active** |
|  |  |  | **NCT03767582** | **CRC/PDAC** | **I/II** | **Completed** |
| **CCR4** | **Mogamulizumab** | **Durvalumab/Tremelimumab** | **NCT02301130** | **Advanced Solid Tumors** | **I** | **Completed** |
|  |  | **Nivolumab** | **NCT02946671** | **Solid Tumor** | **I** | **Completed** |
|  |  |  | **NCT02476123** | **Solid Tumor** | **I** | **Completed** |
| **C5aR** | **IPH5401** | **Durvalumab** | **NCT03665129** | **NSCLC/HCC** | **I** | **Terminated** |
| **IL-1β** | **Canakinumb** | **Spartalizumab** | **NCT04028245** | **RCC** | **Early I** | **Recruiting** |
|  |  |  | **NCT04581343** | **Metastasis PDAC** | **I** | **Active** |
|  |  | **Durvalumab+Chemoradiation** | **NCT04905316** | **Locally Advanced NSCLC** | **II** | **Active** |
| **TNF-α** | **Infliximab** | **Pembrolizumab** | **NCT05034536** | **Metastasis Melanoma** | **II** | **Recruiting** |
| **NSCLC, Non-small-cell carcinoma;** **CRC, Colorectal Cancer;** **PADC, Pancreatic Ductal Adenocarcinomas; HNSCC, Head and Neck Squamous Cell Carcinoma; PC, Pancreatic Cancer; HCC, Hepatocellular Carcinoma; RCC, Renal Cell Carcinoma** | | | | | | |

| **Table S3. Clinical Trials of ICIs Combined with Drugs Targeting Neutrophil Polarization** | | | | | | |
| --- | --- | --- | --- | --- | --- | --- |
| **Target** | **Drug** | **ICIs** | **Trial Number** | **Cancer Type** | **Phase** | **State** |
| **TGF-β** | **NIS793** | **Spartalizumab** | **NCT04390763** | **Metastasis PDAC** | **II** | **Terminated** |
|  | **Livmoniplimab** | **Pembrolizumab** | **NCT06236438** | **NSCLC** | **II/III** | **Recruiting** |
|  |  | **Durvalumab** | **NCT06109272** | **HCC** | **II/III** | **Active** |
| **TGF-β+PD-L1** | **M7824/Bintrafusp alfa** | | **NCT04835896** | **Recurrent/Metastasis GC** | **I/II** | **Completed** |
|  |  |  | **NCT03524170** | **Metastatic Hormone Receptor Positive, HER2 Negative BC** | **I** | **Completed** |
|  |  |  | **NCT03620201** | **Stage II-III HER2 Positive BC** | **I** | **Completed** |
|  |  |  | **NCT05005429** | **Advanced Malignant Pleural Mesothelioma** | **I** | **Completed** |
|  |  |  | **NCT05061823** | **Cancer** | **III** | **Completed** |
|  |  |  | **NCT04428047** | **Operable and Untreated HNSCC** | **II** | **Terminated** |
|  |  |  | **NCT04432597** | **HPV Associated Cancers** | **I/II** | **Active, not recruiting** |
|  |  |  | **NCT04727541** | **Resectable BTC** | **II** | **Terminated** |
|  |  |  | **NCT04481256** | **ESCC** | **Not Applicable** | **Recruiting** |
|  |  |  | **NCT04708067** | **Advanced** **ICC** | **I** | **Active** |
|  |  |  | **NCT04396535** | **Advanced NSCLC** | **II** | **Terminated** |
| **TGF-βR** | **Galunisertib/**  **LY2157299** | **Durvalumab** | **NCT02734160** | **Metastatic PC** | **I** | **Completed** |
|  | **Vactosertib/**  **TEW-7197** | **Durvalumab** | **NCT04893252** | **Stomach Neoplasm** | **II** | **Unknown** |
|  |  |  | **NCT03732274** | **Metastasis NSCLC** | **I/II** | **Completed** |
|  |  | **Pembrolizumab** | **NCT05436990** | **Melanoma** | **I/II** | **Not yet recruiting** |
|  |  |  | **NCT03724851** | **Metastatic CRC or GC** | **II** | **Completed** |
|  |  |  | **NCT03844750** | **CRC/Resectable Hepatic Metastases** | **II** | **Active** |
|  |  |  | **NCT04515979** | **NSCLC** | **II** | **Terminated** |
| **IFN** | **PegIFN-2b** | **Pembrolizumab/Ipilimumab** | **NCT02089685** | **RCC/Melanoma** | **I/III** | **Completed** |
|  |  | **Pembrolizumab** | **NCT02112032** | **Melanoma** | **I** | **Completed** |
|  | **IFN1b** | **Pembrolizumab** | **NCT06212388** | **Melanoma** | **Early I** | **Not yet recruiting** |
|  | **IFN-α2b** | **Ipilimumab** | **NCT01608594** | **Melanoma** | **I** | **Completed** |
| **NSCLC, Non-small-cell carcinoma; CRC, Colorectal Cancer; PADC, Pancreatic Ductal Adenocarcinomas; GC, Gastric Cancer; BC, Breast Cancer; HNSCC, Head and Neck Squamous Cell Carcinoma; BTC, Biliary Tract Cancer; ESCC, Esophageal Squamous Cell Carcinoma; ICC,** **Intrahepatic Cholangiocarcinoma; PC, Pancreatic Cancer; HCC, Hepatocellular Carcinoma; RCC, Renal Cell Carcinoma;** | | | | | | |

| **Table S4. Clinical Trials of ICIs Combined with Drugs Targeting Neutrophil Activation** | | | | | | |
| --- | --- | --- | --- | --- | --- | --- |
| **Target** | **Drug** | **ICI** | **Clinical trial** | **Cancer Type** | **Phase** | **State** |
| **IL-6** | **Tocilizumab** | **Nivolumab/Ipilimumab** | **NCT04940299** | **Advanced Melanoma/NSCLC/UC** | **II** | **Active** |
|  |  | **Atezolizumab** | **NCT04691817** | **Locally Advanced or Metastatic NSCLC Refractory to 1st Line ICI-Based Therapy** | **I/II** | **Recruiting** |
|  |  |  | **NCT03821246** | **Localized Prostate Cancer Prior to Radical Prostatectomy** | **II** | **Active** |
|  |  |  | **NCT03708224** | **HNSCC** | **II** | **Recruiting** |
| **IL-6R** | **Sarilumab** | **Nivolumab/Ipilimumab** | **NCT05428007** | **Stage III/IV Melanoma** | **II** | **Recruiting** |
| **STAT3** | **BBI-608/Napabucasin** | **Nivolumab** | **NCT03647839** | **Metastatic CRC** | **II** | **Completed** |
|  |  | **Ipilimumab/Nivolumab/**  **Pembrolizumab** | **NCT02467361** | **Advanced Cancers** | **I/II** | **Completed** |
|  | **Danvatirsen** | **Pembrolizumab** | **NCT05814666** | **Recurrent/Metastatic HNSCC** | **II** | **Completed** |
| **CSF-1R** | **Axatilimab** | **Durvalumab** | **NCT03238027** | **Solid Tumor** | **I** | **Completed** |
|  | **Cabiralizumab** | **Nivolumab** | **NCT04050462** | **HCC** | **II** | **Completed** |
| **PI3K** | **Duvelisib** | **Nivolumab** | **NCT04688658** | **Unresectable Melanoma** | **I/II** | **Active** |
|  | **GSK2636771** | **Pembrolizumab** | **NCT03131908** | **Melanoma** | **I/II** | **Active** |
|  | **Copanlisib/BAY80−6946** | **Nivolumab** | **NCT03502733** | **Solid tumor/Lymphoma** | **I** | **Active** |
|  |  |  | **NCT04895579** | **NSCLC** | **I** | **Active** |
|  |  |  | **NCT03735628** | **NSCLC / HNSCC /CRC/HCC** | **I/II** | **Completed** |
|  |  | **Durvalumab** | **NCT03842228** | **Solid tumors** | **I** | **Active** |
|  |  | **Nivolumab ± Ipilimumab** | **NCT04317105** | **Solid tumors** | **I/II** | **Active** |
|  | **Eganelisib/IPI-549** | **Nivolumab** | **NCT03980041** | **Advanced UC** | **II** | **Completed** |
|  |  |  | **NCT02637531** | **Advanced Solid Tumors** | **I** | **Unknown** |
|  |  | **Atezolizumab** | **NCT03961698** | **TNBC or RCC** | **II** | **Active** |
| **CD47** | **ALX148/Evorpacept** | **Pembrolizumab** | **NCT03013218** | **Advanced Solid Tumors/Lymphoma** | **I** | **Completed** |
|  |  |  | **NCT05467670** | **OC** | **II** | **Recruiting** |
|  |  |  | **NCT05167409** | **Microsatellite Stable Metastatic CRC** | **II** | **Active** |
|  |  |  | **NCT04675294** | **Advanced HNSCC** | **II** | **Active** |
|  |  |  | **NCT04675333** | **Advanced HNSCC** | **II** | **Active** |
|  | **AO-176** | **Pembrolizumab** | **NCT03834948** | **Solid Tumors** | **I/II** | **Completed** |
|  | **Magrolimab** | **Pembrolizumab** | **NCT06046482** | **Recurrent/Metastatic HNSCC** | **II** | **Terminated** |
|  |  |  | **NCT04854499** | **HNSCC** | **II** | **Terminated** |
| **SIRPα** | **TTI-621** | **Pembrolizumab** | **NCT02890368** | **Relapsed and Refractory Solid Tumors** | **I** | **Terminated** |
|  | **TJ011133** | **Pembrolizumab** | **NCT03934814** | **Solid Tumors/Lymphoma** | **I** | **Completed** |
| **ARG1** | **Pegzilarginase** | **Pembrolizumab** | **NCT03371979** | **SCLC** | **I/II** | **Completed** |
|  | **ADI PEG20** | **Nivolumab ± ipilimumab** | **NCT03922880** | **Uveal Melanoma** | **I** | **Completed** |
|  |  | **Pembrolizumab** | **NCT03254732** | **Advanced Solid Tumors** | **I** | **Terminated** |
| **COX2** | **Celecoxib** | **Nivolumab** | **NCT03864575** | **“Cold” Solid Tumors** | **II** | **Unknown** |
|  |  | **Nivolumab + Ipilimumab** | **NCT03728179** | **Solid Tumors** | **I/II** | **Completed** |
|  |  |  | **NCT03026140** | **Colon Carcinoma** | **II** | **Recruiting** |
|  |  | **Pembrolizumab** | **NCT05756166** | **BC** | **I/II** | **Terminated** |
|  |  |  | **NCT03599453** | **Metastatic TNBC** | **I** | **Completed** |
| **EP2/EP4** | **Grapiprant/ARY-007** | **Pembrolizumab** | **NCT03658772** | **Microsatellite Stable CRC** | **I** | **Completed** |
|  |  |  | **NCT03696212** | **NSCLC** | **I** | **Terminated** |
|  | **OKN4395** | **Pembrolizumab** | **NCT06789172** | **Solid Tumors** | **I** | **Recruiting** |
|  | **TPST-1495** | **Pembrolizumab** | **NCT04344795** | **Solid Tumors** | **I** | **Active** |
| **ILT4** | **MK-4830** | **Pembrolizumab** | **NCT04165083** | **Advanced Solid Tumors** | **I** | **Active** |
|  |  |  | **NCT05342636** | **ESCC** | **I/II** | **Active** |
|  |  |  | **NCT06413095** | **HNSCC/Soft Tissue Sarcoma** | **Early I** | **Completed** |
|  |  |  | **NCT05446870** | **High-grade Serous OC** | **II** | **Completed** |
|  |  |  | **NCT04165096** | **NSCLC** | **II** | **Completed** |
|  |  |  | **NCT04626518** | **RCC** | **I/II** | **Active** |
|  |  |  | **NCT04924101** | **SCLC** | **II** | **Completed** |
|  |  |  | **NCT04938817** | **SCLC** | **I/II** | **Recruiting** |
| **NOS** | **L-NMMA** | **Pembrolizumab** | **NCT03236935** | **NSCLC/UC** | **Ib** | **Active** |
| **NSCLC, Non-small-cell carcinoma; SCLC, Small-Cell Carcinoma; UC, Urothelial Cancer; CRC, Colorectal Cancer; PADC, Pancreatic Ductal Adenocarcinomas; GC, Gastric Cancer; BC, Breast Cancer; HNSCC, Head and Neck Squamous Cell Carcinoma; ESCC, Esophageal Squamous Cell Carcinoma; HCC, Hepatocellular Carcinoma; RCC, Renal Cell Carcinoma; TNBC, Triple Negative Breast Carcinoma; OC, Ovarian Cancer;** | | | | | | |

| **Table S5. Clinical Trials of ICIs Combined with Drugs Targeting NETs** | | | | | |
| --- | --- | --- | --- | --- | --- |
| **Target** | **Drug** | **ICI** | **Trial Number** | **Cancer Type** | **State** |
| **Metformin** | **Metformin** | **Durvalumab** | **NCT03618654** | **HNSCC** | **Completed** |
|  |  | **Nivolumab** | **NCT06884683** | **Metastatic RCC** | **Terminated** |
|  |  | **Pembrolizumab** | **NCT03311308** | **Advanced Melanoma** | **Recruiting** |
|  |  |  | **NCT04414540** | **Metastatic HNSCC** | **Active** |
|  |  |  | **NCT04114136** | **Solid Tumors** | **Recruiting** |
| **TLR9** | **MGN1703** | **Ipilimumab** | **NCT02668770** | **Advanced Cancers** | **Active** |
| **HNSCC, Head and Neck Squamous Cell Carcinoma; RCC, Renal Cell Carcinoma** | | | | | |
